# Comparative transcriptome and metabolite survey reveal key pathways involved in the control of the chilling injury disorder superficial scald in two apple cultivars, ‘Granny Smith’ and ‘Ladina’

**DOI:** 10.3389/fpls.2023.1150046

**Published:** 2023-04-20

**Authors:** Lorenzo Vittani, Francesca Populin, Stefan Stuerz, Andreas Buehlmann, Iuliia Khomenko, Franco Biasioli, Simone Bühlmann-Schütz, Urska Vrhovsek, Domenico Masuero, Angelo Zanella, Nicola Busatto, Fabrizio Costa

**Affiliations:** ^1^ Center Agriculture Food Environment C3A, University of Trento, San Michele all’Adige, Italy; ^2^ Research and Innovation Centre, Fondazione Edmund Mach, San Michele all’Adige, Italy; ^3^ Research Centre Laimburg, Ora, Italy; ^4^ Strategic Research Division Food Microbial Systems, Agroscope, Wädenswil, Switzerland; ^5^ Strategic Research Division Plant Breeding, Agroscope, Wädenswil, Switzerland

**Keywords:** transcriptomics, postharvest, apple, superficial scald, fruit quality, metabolomics, 1-MCP, low oxygen

## Abstract

The low temperature normally applied to prevent fruit decay during the storage of apples, can also triggers the onset of a chilling injury disorder known as superficial scald. In this work, the etiology of this disorder and the mechanism of action of two preventing strategies, such as the application of 1-MCP (1-methylcyclopropene) and storage at low oxygen concentration in ‘Granny Smith’ and ‘Ladina’ apple cultivars were investigated. The metabolite assessment highlighted a reorganization of specific metabolites, in particular flavan-3-ols and unsaturated fatty acids, while the genome-wide transcriptomic analysis grouped the DEGs into four functional clusters. The KEGG pathway and GO enrichment analysis, together with the gene-metabolite interactome, showed that the treatment with 1-MCP prevented the development of superficial scald by actively promoting the production of unsaturated fatty acids, especially in ‘Granny Smith’. ‘Ladina’, more susceptible to superficial scald and less responsive to the preventing strategies, was instead characterized by a higher accumulation of very long chain fatty acids. Storage at low oxygen concentration stimulated a higher accumulation of ethanol and acetaldehyde together with the expression of genes involved in anaerobic respiration, such as *malate*, *alcohol dehydrogenase* and *pyruvate decarboxylase* in both cultivars. Low oxygen concentration, likewise 1-MCP, through a direct control on ethylene prevented the onset of superficial scald repressing the expression of *PPO*, a gene encoding for the polyphenol oxidase enzyme responsible of the oxidation of chlorogenic acid. Moreover, in ‘Granny Smith’ apple, the expression of three members of the VII subgroups of *ERF* genes, encoding for elements coordinating the acclimation process to hypoxia in plants was observed. The global RNA-Seq pattern also elucidated a specific transcriptomic signature between the two cultivars, disclosing the effect of the different genetic background in the control of this disorder.

## 1 Introduction

Fruit quality is the degree of fulfillment of consumers’ expectation and concern a series of intrinsic aspects achieved through the initial process of fruit development and the subsequent ripening syndrome. Due to its role in ensuring an economic success, the fruit quality properties established at harvest need to be maintained and preserved through postharvest storage and shelf-life. Among the several strategies, the lowering of temperature is the primary intervention applied to prevent fruit loss through a controlled delay of fruit ripening (Hawkins, 1922; Hugh Smith, 1958). The fruit wastage occurring between harvest and retail sale is nowadays a first reason undermining food security, leading to severe decay of nutritional value (Bekele, 2021). The low temperature applied to promote postharvest storage, can, however, trigger the onset of typical postharvest disorders assigned to chilling injury phenomenon (Watkins et al., 1995; Cainelli and Ruperti, 2019). Certain apple cultivars such as ‘Delicious’, ‘Fuji’ and ‘Granny Smith’ are susceptible to a typical postharvest disorder known as superficial scald, manifested with the development of dark areas on the first outer layers of the hypodermis cells, making the fruit aesthetically unacceptable, therefore promoting fruit wastage and insecurity (Lurie and Watkins, 2012).

The etiology of superficial scald has been for long associated to the biochemistry of the acyclic sesquiterpene α-farnesene, a volatile organic compound specifically accumulated during ripening in the waxy layer of the fruit cuticle, and exclusively synthesized via the cytosolic mevalonic acid pathway (Lange et al., 2000). α-farnesene can be afterwards down-stream oxidized to conjugated trienols (CTols) and further to 6-methyl-5-hepten-2 one (MHO) (Anet, 1972; Rowan et al., 1995; Rupasinghe et al., 2000; Tsantili et al., 2007). The production of CTols and related oxidation products are also presumably related to an oxidative stress pathway leading to an increased lipid peroxidation enhancing the production of reactive oxygen species (ROS) (Mir et al., 1998; Wang and Dilley, 2000). The onset of superficial scald and the pattern of α-farnesene have also been correlated with internal ethylene concentration, a relationship supported by the ethylene-dependent transcriptional profile of *α-farnesene synthase 1 (AFS1)*, the gene encoding the key enzyme that converts farnesyl diphosphate to α-farnesene (Rupasinghe et al., 2000; Whitaker, 2004). The ethylene-associated mode of action of *AFS1* was further underpinned by the exogenous application of ethylene inhibitors or competitors, such as aminoethoxyvinylglycine (AVG) or 1-methylcyclopropene (1-MCP) (Ju and Curry, 2000; Lurie et al., 2005; Gapper et al., 2006; Busatto et al., 2014), which both down-regulated the mRNA accumulation of this gene. The control of ethylene biosynthesis or signaling, and thus α-farnesene biosynthesis, was also found to be highly effective in preventing the development of superficial scald and is now used as a regular postharvest strategy to control this disorder (Watkins et al., 2000; Shaham et al., 2003; Apollo Arquiza et al., 2005; Busatto et al., 2014). In this regard, also a controlled atmosphere conditions characterized by a low O_2_ and a high CO_2_ concentration prevented the development of scald through a direct control over the production of the hormone ethylene and consequently on the biosynthesis of α-farnesene and the accumulation of CTols, with a parallel increase in the concentration of ethanol in the stored fruits (Lurie and Watkins, 2012).

In addition to the oxidation of α-farnesene, the typical symptoms of superficial scald were also attributed to the oxidation of polyphenolic compounds by the action of the polyphenol oxidase enzyme (PPO). While having an important antioxidant role, phenolic compounds can also contribute to the browning phenomenon. Following chilling injuries, the internal cellular decompartmentalization can promote the oxidative reaction between the PPO enzyme (stored in the plastid) with its substrate chlorogenic acid (stored in the vacuole), leading to the formation of quinones responsible for the dark coloration. Ethylene seems to play a primary role also in this mechanism, since the expression of *MdPPO* gene was either induced by the onset of this hormone or repressed by the exogenous application of its competitor 1-MCP (Busatto et al., 2014; Busatto et al., 2018). Apple skin affected by superficial scald showed an increased concentration of phenolic compounds characterized by antioxidant activity, such as chlorogenic acid, epicatechin, quercetin and procyanidin, scavenging the free radicals produced by the lipid peroxidation (Di Meo et al., 2013). During the oxidation process occurring during this disorder, an early accumulation of phenolic compounds with antioxidant potential have been observed in ‘Granny Smith’ apple (Busatto et al., 2018), as a tentative to counteract the production of ROS, whose homeostasis is regulated by the ROP-GAP rheostat (Zermiani et al., 2015). The rheostatic ROP-GAP control of the H_2_O_2_ homeostasis is a natural adaptation to low oxygen condition, and the genes involved in this process (*MdROPs, MdROP-GEFs, MdROP-GAPs* and *MdRBOHs*) were de-repressed by 1-MCP application, suggesting a negative control of ethylene, which also affected the concentration of glutathione (GSH), one of the most relevant antioxidant for the H_2_O_2_ homeostasis (Noctor and Foyer, 1998; Rahantaniaina et al., 2013). Karagiannis et al. (2020) discovered that 1-MCP can also revert the stimulation of superficial scald symptoms provoked by the application of ozone (O_3_) in storage atmosphere. Through a network based analysis, the authors also identified that the expression of the *PPO* gene was coregulated with the HD-ZIP transcription factor.

It is finally worth noting that most of the investigations carried out to date to elucidate the control of the onset of superficial scald have been mainly focused on the apple cultivar ‘Granny Smith’, being the most susceptible cultivar to this disorder, treated with 1-MCP or antioxidant compounds, such as diphenylamine (DPA) and ethoxyquin (6-ethoxy-2,2,4-trimethyl-1,2-dihydroquinoline) to perturbate the development of this disorder (Whitaker, 2000; Rudell et al., 2005; Jung and Watkins, 2008; Moggia et al., 2010; Karagiannis et al., 2018). To shed light on the regulatory process governing the etiology of superficial scald in apple, we aimed to compare the transcriptome and metabolic re-programming of fruit collected from two apple cultivars, ‘Granny Smith’ (considered as reference) and ‘Ladina’ (a new cultivar highly susceptible to this disorder) stored in regular atmosphere, treated with 1-MCP and maintained in controlled atmosphere (with a low oxygen regime).

## 2 Materials and methods

### 2.1 Fruit harvesting and postharvest storage

To achieve the goals of this survey focused on the understanding of the regulation of the superficial scald in apple, two apple cultivars were employed: ‘Granny Smith’ (GS) and ‘Ladina’ (LA). ‘Granny Smith’ was collected at the experimental orchard of the Research Centre Laimburg (North of Italy), while ‘Ladina’ was collected at the experimental orchard of Agroscope in Wädenswil (Switzerland). Apples from both cultivars were collected from full bearing adult plants at the commercial harvest date, according to standard practice for long-term storage, represented by the assessment of the progression of the fruit firmness and starch degradation, together with historical records. More specifically, fruit of 'Granny Smith' were harvested on the 12^th^ of September with a firmness of 6.8-7.7 kg cm^-2^ and a starch iodine index value of 1.7-2.5 (on a 1 to 5 scale), while for 'Ladina', fruit were instead harvested on the 26^th^ of September with a firmness of 6-7.3 kg cm^-2^ and a starch iodine index value of 6-8.6 (on a 1 to 10 scale). After harvest fruit from both cultivars, were transferred to the lab and divided in three groups: i) stored for 4 months at 1°C with 92% of relative humidity in regular atmosphere (RA); ii) samples treated with 1-methylcyclopropene (1-MCP, applied for 24 hours according to AgroFresh instruction) and stored at regular atmosphere for 7 months at 1°C; iii) apples stored at controlled atmosphere with low oxygen concentration (LO: 0.8-1kPa O_2_ and 1.3 kPa CO_2_) for 7 months at 1°C, conditions already applied in other investigation (Brizzolara et al., 2017). After storage, the level of superficial scald was visually inspected, and portions of skin tissue were sampled from pools of five fruits for each condition and immediately frozen in liquid nitrogen and stored at -80°C for further analysis (metabolite and transcriptome profiling). For each sample three biological replicates were prepared for metabolite and transcriptomic analyses.

### 2.2 Metabolite analytical assessment

The three batches of apples for the two cultivars, ‘Granny Smith’ and ‘Ladina’, respectively, were assessed with different analytical methodologies to profile the concentration of three types of metabolites, such as phenolics, lipids and volatile organic compounds (VOCs).

The phenolic compounds were extracted from 2 grams of frozen skin powder with a solution of water/methanol/chloroform with the proportion 2:4:4, after which followed a second extraction with a 1:2 water/methanol solution, as described in Busatto et al. (2021). The upper phase from the two extractions were combined, brought to 10 mL, and filtered through a 0.2 μm PTFE filter prior to analysis. Separation of the phenolic compounds was achieved on a Waters Acquity HSS T3 column 1.8 μm, 100 mm × 2.1 mm (Milford, MA, USA) with a Water Acquity UHPLC (ultra-performance-liquid-chromatograph) and Xevo TQMS-ESI (Milford, MA, USA), according to the protocol reported in Vrhovsek et al. (2012). Phenolic compounds were quantified as µg g^-1^ of fresh weight (FW).

The lipids profile for the skin tissue of the two apple cultivars was instead obtained using the protocol described in Busatto et al. (2014 and 2021) and originally presented by Della Corte et al. (2015). Lipids were extracted two times with methanol/chloroform (1:2) mixture with the additions of butylhydroxytoluene (BHT, 500mg L^-1^) as antioxidant. Extracted lipids were separated with a Dionex 3000 UHPLC (Thermo Fischer Scientific, Germany) equipped with a RP Ascentis Express column (2.7 µm) and coupled with a triple-quadrupole mass spectrometer API 5500. Each lipid was quantified as µg g^-1^ of FW.

The last aliquot of the same sample set was employed for the characterization of the VOC fingerprinting. The array of volatiles was profiled with a PTR-ToF-MS instrument (Ionicon Analytik GmbH Innsbruck, Austria) through a direct injection and acquisition rate of 1 spectrum/sec. The drift tube condition and sampling protocol was described in Di Pierro et al. (2022). Analytical procedure and data analysis (including Poisson correction, background noise reduction and tentative mass annotation) followed the protocol reported by Cappellin et al. (2011a; 2021b). Within the category of VOCs, the PTR-ToF-MS in ${\text{O}}_{2}{\;}^{+}$ mode was also used to detect the gaseous hormone ethylene. Cappellin et al. (2012) and Costa et al. (2014) previously reported the analytical validity of this tool in accurately profiling the ethylene concentration.

### 2.3 Genome-wide transcriptome analysis

For library preparation and RNA-sequencing, the total-RNA was isolated from three biological replicates/sample/cultivar, using the Spectrum™ Plant Total RNA Kit (Sigma Aldrich, MO, USA). Concentration and purity level of the isolated RNA samples were assessed with both the Nanodrop 8000 Spectrophotometer (Thermo Fischer Scientific, MA; USA) and the Tapestation 2200 (from Agilent Technologies, CA; USA). For library preparation and Illumina sequencing, only samples with a RIN value higher than 7 were used.

The NEB Next Ultra II kit^®^ (BioLabs inc., New England) was used for the preparation of SE RNA-Seq library following the manufacturer’s instruction. Each replicate was obtained from 800 ng of total RNA. Libraries were then quantified using qPCR, and the final quality check was performed with the Agilent D1000 ScreenTape System (Agilent, Santa Clara, CA, USA). Libraries were sequenced using an Illumina NextSeq 500 (read length of 75 bp) at the Functional Genomics Lab at the University of Verona (Italy).

### 2.4 Data analysis

Sequencing reads were analyzed employing the New Tuxedo protocol implemented in CyVerse (Merchant et al., 2016), an on-line freely available pipeline for genomic analysis. Sequences were processed with FastQC, Scythe, Sickle, HiSAT and StringTie for quality check, trimming, sequence alignment on the apple reference genome (assembly ASM211411v1 available at the NCBI database) and transcript quantification, respectively. Transcripts were subsequently analyzed with the R package DESeq2 (Love et al., 2014) for the identification of the set of differentially expressed genes (DEGs) with a foldchange cut-off of 1.5 and FDR ≤ 0.01. DEGs were further used for exploratory data analysis carried out with iDEP, an integrated web application for the transcript and pathway analysis (Ge et al., 2018). Gene and pathway enrichment analysis, based on Gene Ontology (GO) and KEGG database, was performed with ShinyGO v0.76 on-line tool (Ge et al., 2020) filtering for FDR ≤ 0.05.

The number of clusters identified through the heatmap analysis was established employing the K-means clustering approach, for the identification of the most reliable number of subgroups of observation through the computation of a distant matrix of (dis)similarity. The determination of the optimal numbers of clusters within the dataset was based on the Average Silhouette method, with *a priori* defined number of K-mean clustering groups set from 2 to 10. The K-means cluster analysis was carried out with tidyverse, cluster and factoextra R packages (Wickham et al., 2019), implementing the Silhouette method for the determination of the optimal number of clusters. The multivariate statistical procedure of Principal Component Analysis (PCA) for the orthogonal data structure analysis employed to summarize the variability contained in the metabolite and transcriptomic dataset was carried out with the ‘prcomp’ function of tidyverse R package (Wickham et al., 2019).

To perform a Pearson-correlation based interactome between the gene expression profile and the accumulation pattern of the most significant metabolites, a reduced number of DEGs was selected from the original dataset. To this end, a Scree test was carried out to identify the number of factors to retain in the PCA performed on the DEGs (Cattell, 1966), on which the most relevant numbers of variables (DEGs) explaining the highest quote of variability was selected. The Scree test and the selection of the most important contributing variables were carried out with ‘fviz_screeplot’ ‘fviz_contrib’ functions of factoextra R package (**Supplementary Figure 1**).

The DEGs-metabolites interactome was computed with the R package ‘broom’. Correlation showing a P-value ≤ 0.05 and interval included between -1/-0.8 (for negative correlation) and 1/0.8 (positive correlations) were shown with a circular plot generated with the Circus software (Krzywinski et al., 2009).

Venn diagram was created with the on-line application Venny 2.1 (https://bioinfogp.cnb.csic.es/tools/venny/index.html).

## 3 Results

### 3.1 Fruit of ‘Granny Smith’ and ‘Ladina’ showed a different incidence of the superficial scald disorder

Fruit of both cultivars were stored following three postharvest strategies. The first postharvest storage condition was characterized by only controlling the temperature set at 1°C (RA), which promoted the development of the superficial scald symptoms, with a percentage of affected fruit of about 70 and 100% for ‘Granny Smith’ and ‘Ladina’ apple cultivars, respectively. The other two strategies, employed to prevent this disorder, were represented by the cold storage at a regular atmosphere but with fruit treated with the ethylene competitor 1-MCP and by cold storage in controlled atmosphere at ultra low concentration of oxygen (LO) of about 1 kPa. While both strategies resulted to be highly effective in controlling the development of superficial scald in ‘Granny Smith’ apples, bringing the level of scald incidence down to 1%, ‘Ladina’ showed a different response. In this cultivar, in fact, both 1-MCP and LO did not fully prevent the onset of superficial scald as observed for ‘Granny Smith’, which remained at the level of 20% and 9% of affected fruit for the two strategies, respectively (**Supplementary Figure 2**).

### 3.2 Phenolic profiling reveals a specific metabolic composition for ‘Granny Smith’ and ‘Ladina’ apples

**Figure 1:**
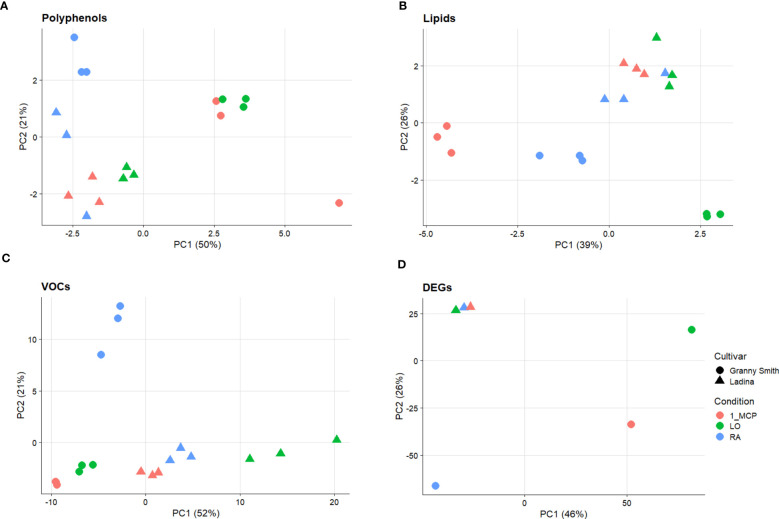
Principal Component Analysis (PCA) performed on polyphenols **(A)**, lipids **(B)**, volatile organic compounds (VOCs) **(C)** and differentially expressed genes (DEGs) **(D)** in ‘Granny Smith’ (●) and ‘Ladina’ (▲) apple cultivars. RA= Regular Atmosphere (●); 1-MCP= Regular Atmosphere + 1-MCP treatment (●); LO= Low Oxygen condition (●). In the PCA of panel **(A–C)** the distribution of the three biological replicates is reported.

**Figure 2:**
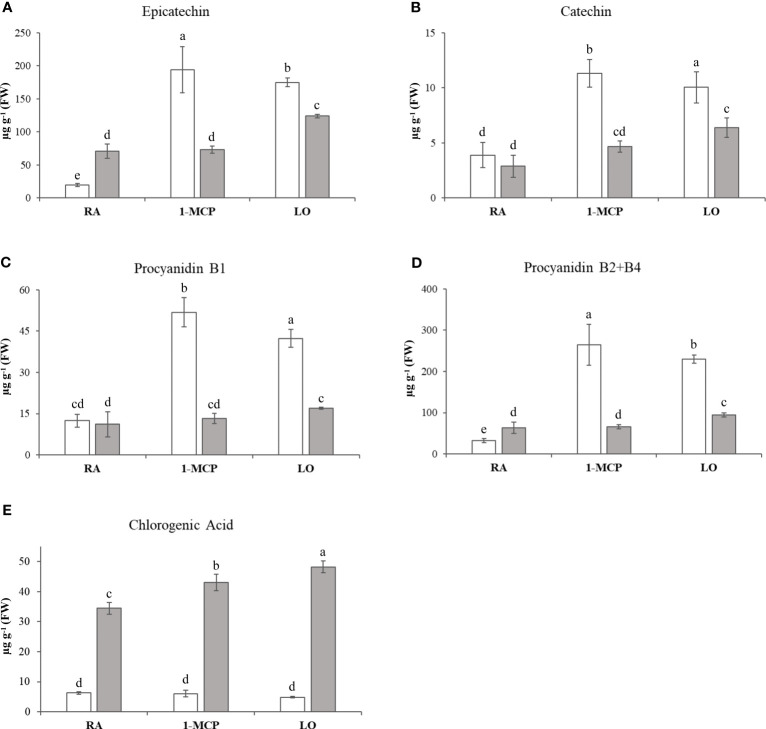
Flavan-3-ols quantification in ‘Granny Smith’ (withe bar) and ‘Ladina’ (grey bar). **(A)** epicatechin, **(B)** catechin, **(C)** procyanidin B1, **(D)** procyanidin B2+B4 and chlorogenic acid **(E)**. The concentration is expressed as µg·g^-1^ of fresh weight (FW). The error bars correspond to the standard deviation. RA, Regular Atmosphere; 1-MCP, Regular Atmosphere + 1-MCP treatment; LO, Low Oxygen condition. Fisher’s least significant difference (LSD) test was applied, p-value ≤ 0.01. Different letters indicate difference statistically significant.

The overall array of phenols, represented by 17 compounds (**Supplementary Table 1**), was profiled for the three sets of samples/cultivar, and their variability was employed to plot the distribution of each sample over a 2D-PCA plot defined by the first two principal components, representing together 71% of the total phenolic variability (**Figure 1A**). The distinct contribution of the first two principal components clearly distinguished the efficacy of the treatment in the control of superficial scald from the effect of the genetic background of the two cultivars. PC1, accounting for 50% of the total variance, clustered, in the positive quadrant, the two samples of ‘Granny Smith’ in which the development of superficial scald was totally prevented by both the application of 1-MCP (GS_1-MCP) and LO (GS_LO), while the rest of the samples were projected in the negative area of the plot. The PC2, accounting for 21% of the total variance, was instead oriented to discriminate the two cultivars. The GS samples were located in the positive area of the PC2 quadrant, while the three symptomatic samples of ‘Ladina’ were positioned towards the negative area of PC2. The three groups of samples (1: GS_1-MCP, GS_LO; 2: GS_RA and 3: LA_RA/1-MCP/LO), as defined by the PCs coordinates, were distinguished by the pattern of specific phenolic compounds, as illustrated by the loading plot (**Supplementary Figure 3A**). The first group was in fact characterized by a higher concentration of *cis*-piceide, phlorizin, catechin, epicatechin, procyanidin B1, procyanidin B2+B4 (as B2), quercetin-3-glucoside+quercetin-3-galactoside (as qercetin-3-glucoside), rutin and arbutin, while in group 2 and 3 a higher concentration of vanillin, chlorogenic, neochlorogenic and cryptochlorogenic acids, coniferyl alcohol and kaempferol-3-rutinoside, was observed. From the overall array of phenolic compounds, the group of flavan-3-ols (catechin, epicatechin, procyanidin B1 and procyanidin B2+B4) were generally more accumulated in ‘Granny Smith’ than ‘Ladina’, especially in asymptomatic samples (GS_1-MCP and GS_LO), with an average fold change (GS/LA) of 3.33 and 1.89 for 1-MCP and LO respectively (**Figures 2A–D**).

In ‘Granny Smith’, moreover, the accumulation of these compounds was higher in the 1-MCP treated sample, while in ‘Ladina’ the highest accumulation was observed in the LO sample, although the absolute values were lower than in ‘Granny Smith’. The three samples of the cultivar ‘Ladina’ were instead distinguished by a higher accumulation of chlorogenic acid, which increased from RA to 1-MCP and LO samples, respectively (**Figure 2E**).

### 3.3 Characterization of lipid compounds between the two apple cultivars

**Figure 3:**
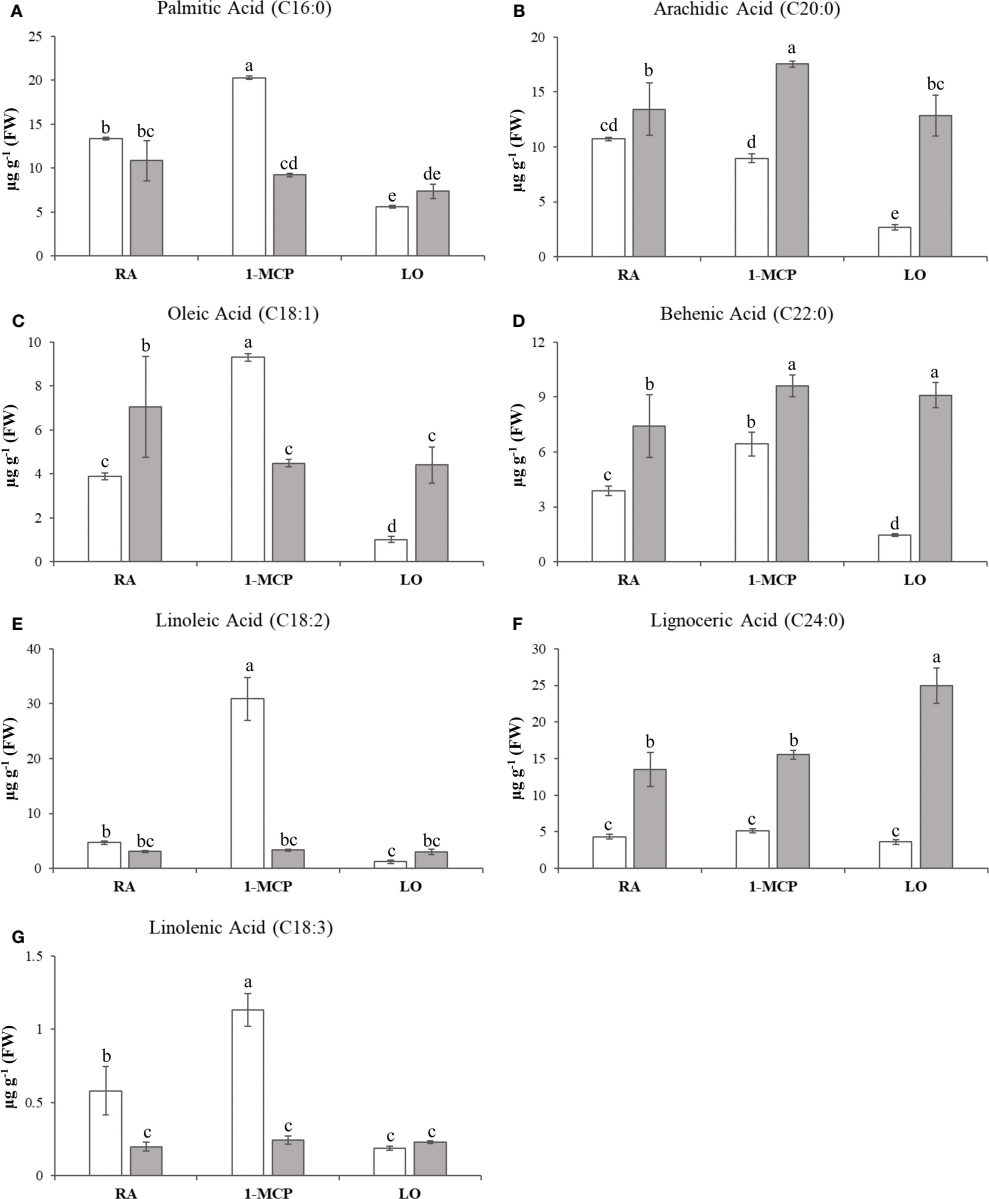
Lipids quantification in ‘Granny Smith’ (white bar) and ‘Ladina’ (grey bar) apple cultivars. **(A)** palmitic acid, **(B)** arachidic acid, **(C)** oleic acid, **(D)** behenic acid, **(E)** linoleic acid, **(F)** lignoceric acid and **(G)** linolenic acid. The concentration was express as µg·g^-1^ of fresh weight (FW). The error bars correspond to the standard deviation. RA, Regular Atmosphere; 1-MCP, Regular Atmosphere + 1-MCP treatment; LO, Low Oxygen condition. Fisher’s least significant difference (LSD) test was applied, p-value ≤ 0.01. Different letters indicate difference statistically significant.

The sample set defined in the experimental scheme of this survey was also employed for a comprehensive characterization of lipids. The variability of the 15 lipidic compounds (**Supplementary Table 2**) enabled the distribution of the six samples over a 2D-PCA score plot defined by the first two PCs, accounting together for the 65% of the total variability (**Figure 1B**). Regarding the composition of lipids, the distinction between the two cultivars was mainly assigned to the orientation of the PC2 variable (explaining 26% of the total variability), with ‘Ladina’ and ‘Granny Smith’ plotted in the positive and negative quadrant of the plot, respectively. The distribution of the samples based on the lipid profile highlighted a distinct behavior of the two apple cultivars. While the three samples of ‘Ladina’ were closely clustered, the samples of ‘Granny Smith’ were more spread over the entire area of the plot. The GS_RA sample was in fact located in the central area, in proximity to the cluster of ‘Ladina’, while the other two samples, characterized by an asymptomatic phenotype, were instead oppositely positioned. More specifically, the sample stored at low oxygen concentration was oriented towards the positive area of the PC1, while the sample treated with 1-MCP was oppositely located in the negative PC1 area. The inspection of the lipid loading plot (**Supplementary Figure 3B**) revealed that the linear distribution of the ‘Granny Smith’ samples from LO to RA and 1-MCP was associated to a continuous increase of the entire array of lipids. The application of 1-MCP in ‘Granny Smith’ showed, moreover, a specifically increased accumulation of unsaturated fatty acids (UFAs), in particular oleic acid (C18:1), linoleic acid (C18:2) and linolenic acid (C18:3), together with the saturated fatty acid palmitic acid (C16:0) (**Figures 3A, C, E, G**). An exception was observed for the arachidic acid (C20:0) that was more stimulated by the application of 1-MCP in ‘Ladina’ rather than in ‘Granny Smith’ (**Figure 3B**). ‘Ladina’, moreover, showed a higher accumulation of other saturated very long chain fatty acids (VLCFAs) when compared to ‘Granny Smith’, such as behenic acid (C22:0) and lignoceric acid (C24:0) (**Figures 3D, E**).

### 3.4 Volatilome profiling unravel as the emission of VOCs was affected by the storage strategies in a cultivar dependent fashion

**Figure 4:**
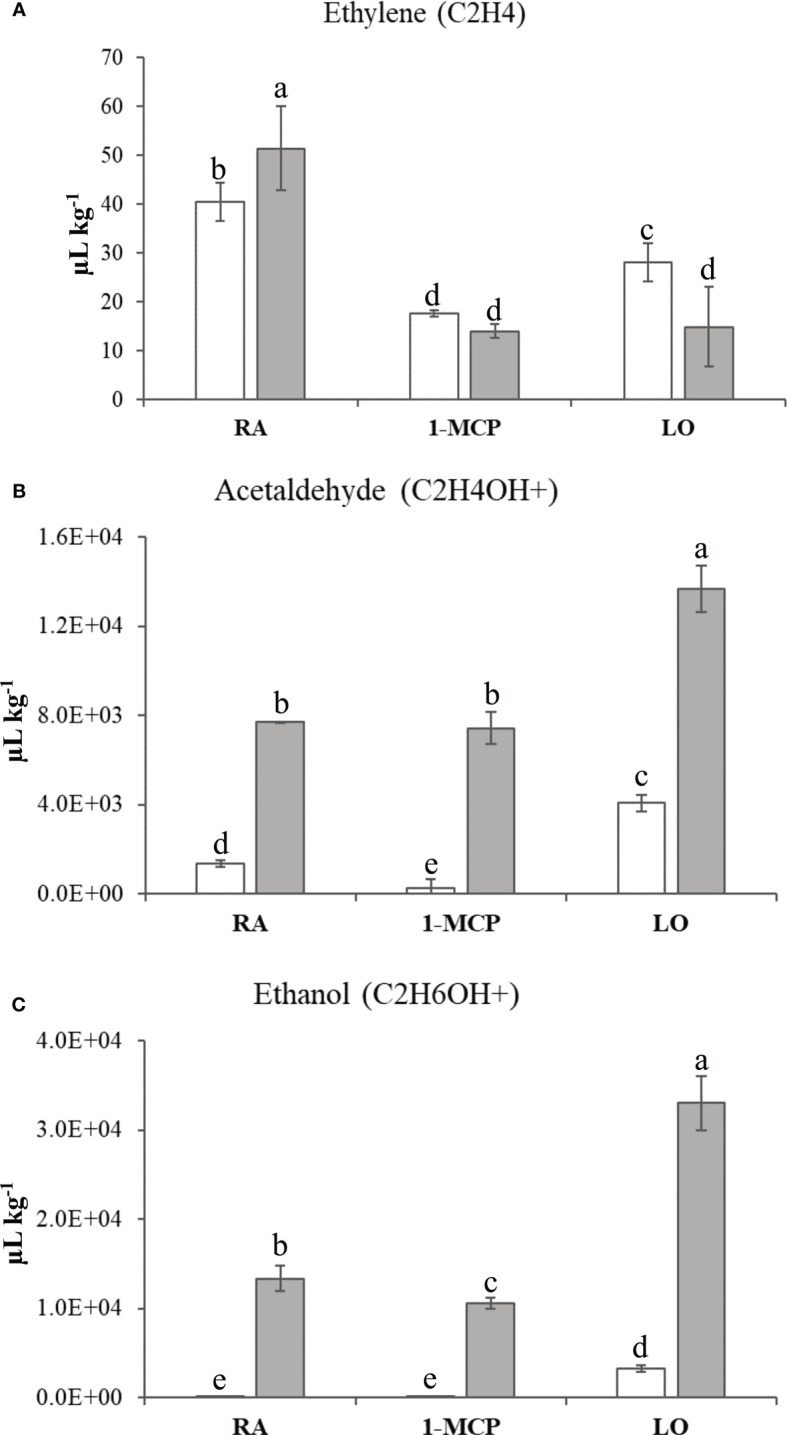
Ethylene **(A)**, Acetaldehyde **(B)** and Ethanol **(C)** quantifications in ‘Granny Smith’ (withe bar) and ‘Ladina’ (grey bar) apple cultivars. The concentration was express as µL·kg^-1^. The error bars correspond to the standard deviation. RA, Regular Atmosphere; 1-MCP, Regular Atmosphere + 1-MCP treatment; LO, Low Oxygen condition. Fisher’s least significant difference (LSD) test was applied, p-value ≤ 0.01. Different letters indicate difference statistically significant.

The volatilome, represented by 106 mass peaks (**Supplementary Table 3**), detected by PTR-ToF-MS, allowed the distribution of the samples over a 2D-PCA plot defined by the first two principal components accounting together for 73% of the VOC variability (**Figure 1C**; **Supplementary Figure 3C**). The PC1 (explaining 52% of the total variability) clearly distinguished the two cultivars, while the PC2 (21% of variability explained) was more effective in the characterization of the type of storage employed. Especially for ‘Granny Smith’, while the control sample was plotted in the PC2 positive area, the two asymptomatic samples (1-MCP and LO) were located in the negative PC2 area of the plot. The samples of ‘Ladina’ showed instead a different behavior, being more closely clustered in the PC1 positive and PC2 negative quadrant of the plot. Among the several volatiles, it is worth to underline the accumulation pattern of three specific compounds. Ethylene, for both cultivars, showed a higher accumulation in the control samples (RA) with regards to 1-MCP and LO, underlining the efficacy of both strategies in interfering with the physiological production of this hormone (**Figure 4A**). The other two VOCs were specifically related to fermentative metabolites, such as acetaldehyde and ethanol (**Figures 4B, C**). For both cultivars, the two metabolites showed a similar pattern, with a high accumulation in the sample stored at low oxygen. However, all the three samples of ‘Ladina’ showed a much higher accumulation of these metabolites compared to ‘Granny Smith’, with a fold change spanning from 3.36 to 27.90 for acetaldehyde and from 10.13 to 356.34 for ethanol.

### 3.5 Genome-wide transcription comparison between two apple cultivars

**Figure 5:**
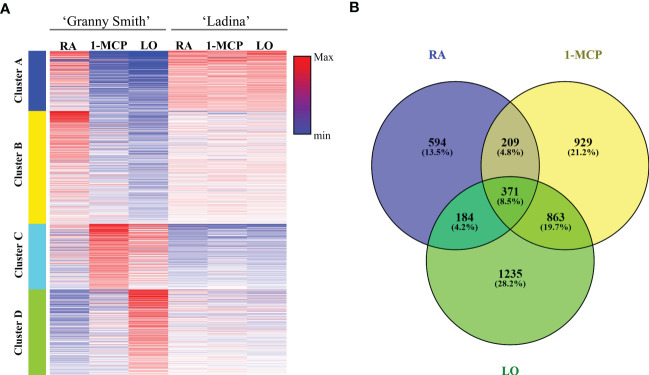
Heatmap **(A)** showing the expression profile of DEGs in ‘Granny Smith’ and ‘Ladina’. K-mean cluster analysis was also performed, and four different clusters were identified (Cluster A–D). RA, Regular Atmosphere; 1-MCP, Regular Atmosphere + 1-MCP treatment; LO, Low Oxygen condition. In **(B)** is reported the Venn diagram of the DEGs identified between the two cultivars for each storage strategies employed.

The genome-wide RNA-seq analysis produced an average of 35,663,394 and 39,172,236 base sequenced for ‘Granny Smith’ and ‘Ladina’ respectively, aligned over the genome freely available at the NCBI database. From the total number of 57,423 genes, 6100 resulted statistically significant (DEGs) across the profile of the two apple cultivars. The expression pattern of the final set of DEGs was initially exploited to depict the sample distribution over a 2D-PCA plot (**Figure 1D**). Consistent with the observation made for the multivariate analysis carried out for the three classes of metabolites, also for the transcription analysis the two cultivars were clearly separated. More specifically, the PC1 (accounting for 46% of the total transcriptome variability) distinguished the symptomatic samples (the three samples of ‘Ladina’ and GS_RA, plotted on the negative part of the PC1 area) from the asymptomatic ones (GS_1-MCP and GS_LO, plotted in the positive PC1 area). The PC2, although explaining a lower quote of variance (26%), distinguished the two cultivars, with an exception. While GS_RA and GS_1-MCP samples were located in the negative area of the PC2, the three samples of ‘Ladina’, together with GS_LO, were located in the positive area of the PC2 although the latter was plotted on the opposite side of the plot. The transcription pattern of DEGs was furthermore organized into a heatmap which clearly depicted four clusters based on the K-mean clustering (**Figure 5A** and **Supplementary Figure 4**; **Supplementary Table 4**). The cluster A (1160 DEGs) was mainly represented by DEGs expressed in the four symptomatic samples expressing symptoms of superficial scald, such as GS_RA, and the three samples of ‘Ladina’ (LA_RA, LA_1-MCP and LA_LO). The cluster B was instead composed by 2058 DEGs specifically overexpressed in RA sample of ‘Granny Smith’ and to a lower extent in the three samples of ‘Ladina’. Clusters C and D, including 1346 and 1536 DEGs, respectively, showed instead a particular expression profile. Both clusters were in fact distinguished by genes more expressed in asymptomatic fruit of ‘Granny Smith’, but with a specific behavior. While the DEGs of cluster C were more expressed in skin of fruit treated with 1-MCP, the differentially expressed genes included in cluster D were mainly represented by elements induced by the storage at low oxygen concentration.

### 3.6 KEGG pathways and GO enrichment analysis of the four functional K-clusters

**Figure 6:**
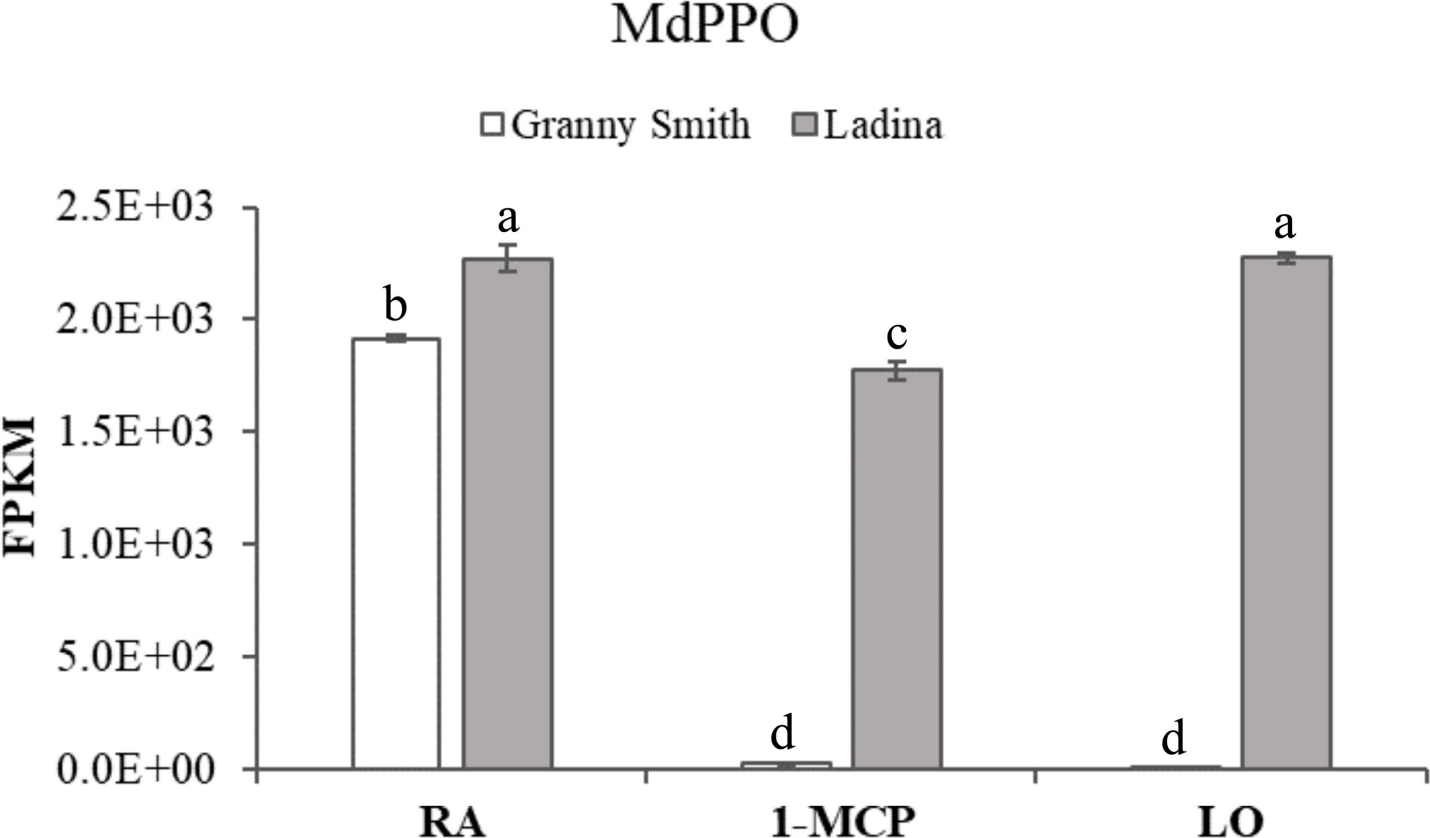
*Polyphenol oxidase (PPO)* expression level in ‘Granny Smith’ and ‘Ladina’ apple cultivars. The error bars correspond to the standard deviation. RA, Regular Atmosphere; 1-MCP, Regular Atmosphere + 1-MCP treatment; LO, Low Oxygen condition. FPKM, fragments per kilobase of transcript per million fragments mapped. Different letters indicate difference statistically significant according to Fisher’s least significant difference (LSD) test, p-value≤0.01.

The total list of DEGs plotted in the heatmap (**Figure 5A**) were further exploited to interrogate the KEGG database and to perform a GO enrichment analysis on the biological process dictionary in order to gain knowledge about the functional pathways and biological process involved in the control of this disorder. The KEGG analysis for the genes included in the cluster A identified 18 KEGG pathways (**Supplementary Table 5**) and 113 gene ontologies (**Supplementary Table 6**). The most important and enriched pathways were represented by the biosynthesis of secondary metabolites, glutathione metabolism, terpenoid backbone synthesis, arachidonic acid metabolism and monoterpenoid biosynthesis. The GO enrichment consistently revealed the identification of processes related to oxidative-reduction process, glutathione metabolism, response to lipids, defense response, response to stress, lipid biosynthesis and homeostasis, and cellular response to cold. The cluster B, characterized by 29 KEGG pathways (**Supplementary Table 7**) and 136 GO ontologies (**Supplementary Table 8**) showed similarities with regards to cluster A, being represented by DEGs showing a higher expression in GS_RA but a reduced transcript accumulation in the three samples of ‘Ladina’. For the KEGG analysis, the most enriched pathways were represented by pyruvate, glycolysis/gluconeogenesis and citrate cycle (TCA cycle) metabolism, together with glutathione metabolism. The most enriched GO terms were instead related to oxidation-reduction process, cellular respiration, aerobic respiration, lipid metabolism and response, and cellular catabolic processes, *i.e.* lipid, carbohydrate, carboxylic and monocarboxylic acid. Specifically in cluster B, it was also identified a GO enrichment related to secondary metabolite processes and included in the KEGG pathway of the phenylpropanoid biosynthesis. With regards to this group, three elements annotated as *phenylalanine ammonia-lyase (PAL)* (LOC103450046, LOC103423368 and LOC103430265; **Table 1**) showing an expression level much higher in the symptomatic samples (GS_RA, LA_RA, LA_1-MCP and LA_LO) when compared to the asymptomatic (GS_1-MCP and GS_LO) were retrieved, with an average fold change of 35.13, 38 and 14.7, respectively (**Supplementary Figure 5**). Phenylalanine ammonia-lyase is the first enzyme committed in the phenylpropanoid pathway for the biosynthesis of phenolic compounds. This pattern was furthermore consistent with the *chloroplastic polyphenol oxidase* gene (*MdPPO*; **Table 1**), whose expression was 141 times higher in the tissues of the symptomatic samples with regards to the two asymptomatic ones (**Figure 6**).

**Table 1:** Most relevant DEGs discussed in the manuscript, reporting, for each gene, the ID, the annotation and the chromosome.

Gene ID	Gene Annotation	Chromosome
LOC103450046	Phenylalanine ammonia-lyase 1	12
LOC103423368	Phenylalanine ammonia-lyase 1-like	12
LOC103430265	Phenylalanine ammonia-lyase 1	1
MdPPO	Polyphenol oxydase	10
LOC103451162	Phospholipase A(1) LCAT3-like	12
LOC103412148	Phospholipase D delta-like	10
LOC103422578	13-Lipoxygenase 2-1 chloroplastic-like	11
LOC103432769	Alcohol dehydrogenase 1	4
LOC103435799	Alcohol dehydrogenase-like	5
LOC103400528	Alcohol dehydrogenase 1-like	15
LOC103408309	Malate dehydrogenase	2
LOC103402801	Malate dehydrogenase	16
LOC103424543	Malate dehydrogenase, chloroplastic-like	14
LOC108171114	Malate dehydrogenase, chloroplastic-like	14
LOC103425929	Pyruvate decarboxylase 2-like	3
LOC103425939	Pyruvate decarboxylase 2-like	3
LOC103408131	Ethylene-responsive transcription factor ERF073-like	3
LOC103432615	Ethylene-responsive transcription factor ERF073-like	3
LOC103449153	Ethylene-responsive transcription factor ERF071-like	11

**Figure 7:**
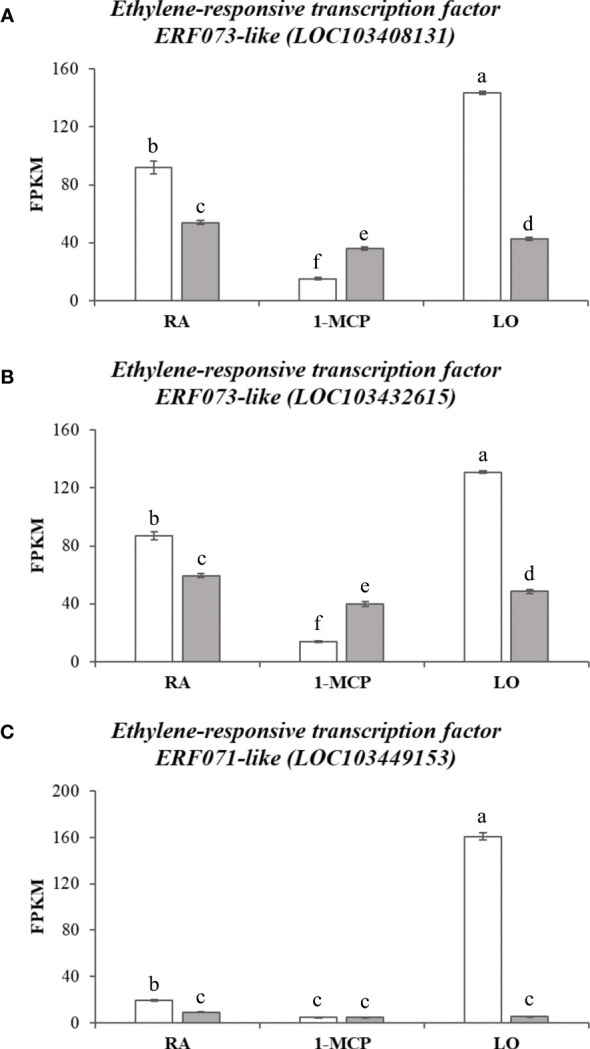
Expression level in ‘Granny Smith’ (white bar) and ‘Ladina’ (grey bar) apple cultivars of three *Ethylene-responsive transcription factor (ERF-like)* genes depicted in **(A–C)**, respectively. The error bars correspond to the standard deviation. RA, Regular Atmosphere; 1-MCP, Regular Atmosphere + 1-MCP treatment; LO, Low Oxygen condition. FPKM, fragments per kilobase of transcript per million fragments mapped. Different letters indicate difference statistically significant.

Clusters C and D, characterized by gene expression in asymptomatic samples following to the treatment with 1-MCP or storage at low oxygen, showed instead a substantial difference in terms of pathway and gene enrichment analyses. Cluster C (represented by 14 KEGG pathways and 72 GO terms; **Supplementary Tables 9**, **10**) was mainly distinguished by pathways involved in the biosynthesis and metabolism of fatty acids and cutin biosynthesis. The GO term identified an enriched cluster associated to lipids and fatty acids metabolic process as well as the processing of very long chain fatty acids, cutin, wax and response to cold. Within this cluster, for instance, two *phospholipase members* of both types, *A-like* (LOC103451162) and *D-like* (LOC103412148) were identified, with a higher expression in ‘Granny Smith’ than ‘Ladina’ (with a fold change of 13.4 and 20.7, respectively) and, more specifically, in the GS_1-MCP sample (**Supplementary Figure 6** and **Table 1**). The phospholipases are a category of enzymes that hydrolyzing phospholipids into fatty acids coordinate an important lipid remodeling essential to stimulate an immediate response to environmental stresses. In the same cluster C, another DEG related to a *13-lipoxygenase 2-1 chloroplastic-like* (LOC103422578) (**Supplementary Figure 6** and **Table 1**), with an expression in GS_1-MCP 185 times higher in ‘Granny Smith’ than in ‘Ladina’, was identified. Lipoxygenases belong to a family of enzymes catalyzing the deoxygenation of polyunsaturated fatty acids and hereby playing a relevant role in their biosynthetic process. The last cluster D (22 KEGG pathways, **Supplementary Table 11**, and 91 GO, **Supplementary Table 12**) grouped the DEGs expressed during storage in hypoxia condition in GS. Most of the enriched pathways were in fact related to the photosynthetic pathway system, carbon metabolism, glycolysis, pyruvate metabolism and amino acids metabolism. For the GO terms, the most enriched processes were consistently represented by elements related to photosynthesis, REDOX process, metabolism and response to ROS stress related to anoxia and hypoxia conditions and ethylene signaling pathway. The activation of the fermentative processes necessary to produce ATP, was also confirmed by the high expression of genes related to *alcohol dehydrogenase* (LOC103432769, LOC103435799 and LOC103400528), *malate dehydrogenase* (LOC103408309, LOC103402801, LOC103424543 and LOC108171114) and *pyruvate decarboxylase* (LOC103425929 and LOC103425939) identified in this cluster (**Supplementary Figure 7** and **Table 1**). In this context, within the enriched GO processes, a cellular response to anoxia was identified. This group of GO was also represented by three genes (LOC103408131, LOC103432615 and LOC103449153; **Table 1**) belonging to the *ERF073* and *071* class and showing an expression level much higher in the GS_LO with regards to the other samples, with an average fold change of 2.9, 2.6 and 18.7, respectively (**Figure 7**). A blast analysis reveal that these genes are ortholog of the *Arabidopsis At1g53910* (e-value of 6e^-28^), the *RAP2.12* gene belonging to the VII subgroup of *ERF* transcription factor functioning as an oxygen sensing mechanism (Giuntoli and Perata, 2018). The set of DEGs identified between the two apple cultivars for each storage strategies, was also visualized through a Venn diagram that highlighted unique and common genes differentially expressed among the different comparisons (**Figure 5B**). In this grouping, it is worth noting the core set of 371 DEGs common to all the three pair-wise analyses. The GO analysis of this group identified 29 enriched GO terms (P-value ≤ 0.01), among which the most relevant were represented by oxidation-reduction process, glutathione metabolic process, response to alcohol, defense response, response to oxygen-containing compounds and cellular response to hypoxia.

### 3.7 Transcriptome-metabolite correlation-based interactome

**Figure 8:**
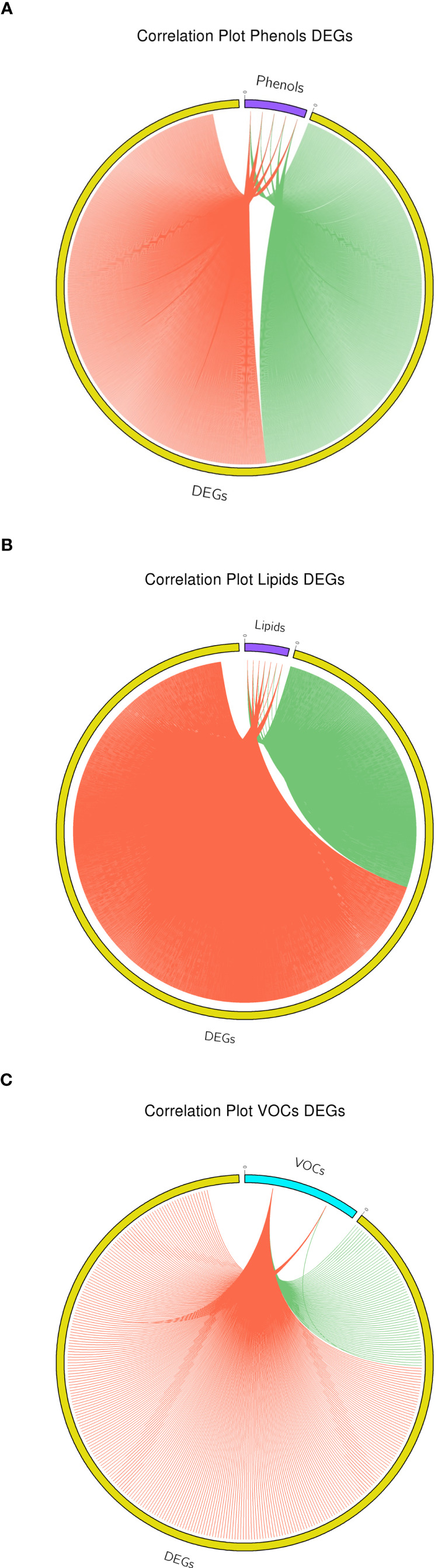
Interactome based correlation between DEGs and phenolic compounds **(A)**, lipids **(B)** and two volatiles, acetaldehyde and ethanol **(C)**. For each plot, positive and negative correlation are highlighted with red and green color, respectively.

The transcriptome-metabolite interactome was carried out computing the Pearson correlation between 3262 DEGs, representing the most relevant and important variables describing the highest quote of variability, and the set of metabolites differentially accumulated between the two apple cultivars. For the phenols, 5 compounds (illustrated in **Figure 2**) were employed in the interactome analysis with DEGs (**Supplementary Table 13**). The phenolic-based interactome showed a more equal proportion between positive and negative correlations, contributing for the 53.77% (1834) and 46.23% (1577) of the total interactome, respectively (**Figure 8A**). Within this interactome, chlorogenic acid, and procyanidin B1 were distinguished by a higher number of positive correlations, with a fold change spanning from 1.3 to 3.8. On the contrary, epicatechin was distinguished by a higher number of negative correlations, with a fold change of 2.15. The KEGG analysis of the phenolic-based interactome identified the pathways related to glutathione, pyruvate, terpenoid and phenylalanine as the most enriched metabolisms. These pathways were further validated by the GO enrichment analysis that identified the cellular response to hypoxia as the most enriched GO term. For the lipids, the interactome was computed with 7 compounds (arachidic acid, behenic acid, lignoceric acid, linoleic acid, linolenic acid, oleic acid and palmitic acid; illustrated in **Figure 3**), identifying 3437 correlations. The lipids showed 72.2% (2481) positive correlations and 27.8% (956) negative correlations (**Figure 8B**). Despite the phenols, almost all the lipid compounds were characterized by a higher proportion of positive correlation, beside arachidic acid (**Supplementary Table 14**). For the lipid-based interactome, both KEGG and GO identified as the most enriched processes were related to fatty acid metabolism, biosynthesis, elongation and biosynthesis of very long chain fatty acids. From the entire array of VOCs, two volatiles (acetaldehyde and ethanol) were considered in the interaction with the DEGs, identifying a total of 340 interactions. Both showed a higher proportion of positive correlations (**Supplementary Table 15**), with a fold change over the negative of 3.75 and 68 for acetaldehyde and ethanol, respectively (**Figure 8C**). The GO analysis performed on the core set of DEGs employed in the VOC-interactome identified processes related to biosynthesis of secondary alcohols and regulation of response to oxidative stress.

## 4 Discussion

In this work we have investigated the effect of both, the genetic background of two apple cultivars and two storage strategies towards the prevention of the superficial scald in apple.

### 4.1 The development of superficial scald is associated with a metabolic transition of specific phenolic compounds

The metabolic profiling carried out to analyze the concentration of phenylpropanoids across the samples included in this study underlined important differences related to the genetic background of ‘Granny Smith’ and ‘Ladina’. The PCA distribution elucidated that the two postharvest strategies employed to prevent the development of superficial scald, represented by the treatment with 1-MCP and the controlled atmosphere, modified the composition of this class of metabolites in the two asymptomatic samples of ‘Granny Smith’. In GS_1-MCP and GS_LO samples, the levels of catechin, epicatechin, procyanidin B1 and B2+B4 were much higher than in the other samples (**Figures 2A–D**). The role of catechin and epicatechin in asymptomatic fruit was also observed during the onset of another chilling injury phenomena, the soggy breakdown in ‘Honeycrisp’ apple cultivar (Leisso et al., 2015), where the symptoms shown by the affected tissues were associated to the polymerization reaction catalyzed by the PPO, while the asymptomatic tissues were characterized by a higher accumulation of phenolic compound with a protecting and ROS scavenging role (Pourcel et al., 2007). Most of the phenolic compounds are indeed characterized by a double functionality. At low concentration they function as antioxidant through different mechanisms, such as free radical scavenging, hydrogen donor or single oxygen quenching, while at higher concentration they promote oxidative reactions (Robards et al., 1999). The accumulation pattern of the phenolic compounds observed in this study can be thus attributed to these mechanisms. The higher concentration observed for these four phenolic compounds in the asymptomatic samples (GS_1-MCP and GS_LO) contrary-reflected the expression pattern of the *MdPALs* and *MdPPO* genes (**Figure 6** and **Supplementary Figure 5**). The reduction in expression level of *MdPPO* in these two samples highly reduced the polymerization catalysis of these phenolic compounds, which are therefore more accumulated and thus playing a higher protecting role. This is also justified by the finding that among the array of phenolic compounds assessed here, the asymptomatic samples, GS_1-MCP and GS_LO, were characterized by an increased accumulation of two flavan-3-ols (catechin and epicatechin, known to have a greater antioxidant activity) and two condensed tannins, procyanidin B1 and B2+B4 (Meyer et al., 1998; Robards et al., 1999). It is also worth noting that the phenolic based interactome identified that *MdPPO* was negatively correlated with catechin, procyanidin B1 and B2+B4, with correlation value spanning from -0.94 (p-value: 0.006) to -0.96 (p-value: 0.002). Procyanidin B-type also plays important antioxidant role, by inhibiting the peroxidation of lipids (Rue et al., 2018; Rauf et al., 2019). On the contrary, the samples of the cultivar ‘Ladina’, more susceptible and prone to develop the superficial scald disorder, were distinguished by a higher (7.3 fold change) concentration of chlorogenic acid (**Figure 2E**), the preferred substrate for the action of the polyphenol oxidase enzyme in pome fruit and known to react with PPO for the generation of melanin, responsible for the typical dark coloration occurring during the onset to this disorder (Amiot et al., 1992). The accumulation pattern in both cultivars did not show any relevant modulation regarding the type of treatment, therefore, the difference observed had to be solely attributed to the genetic difference between these two cultivars. This hypothesis was furthermore validated by the 5.46 fold change difference of chlorogenic acid at the level of the two RA samples, both symptomatic for this disorder.

### 4.2 The ethylene competitor 1-MCP stimulate a cultivar-dependent fatty acid reprogramming towards an improved resistance to chilling injury disorder

Amongst the several disturbances provoked by low temperatures, a modification of the plant membranes, particularly in plastids, is one of the most important events. Plant membranes are mainly composed by building blocks of glycerophospholipids, which are made of glycerophosphoric acid linked to fatty acids (He and Ding, 2020; Zhukov and Shumskaya, 2020). Cold temperatures induce important modifications in the membrane, contributing to a rigidification of the structure (He et al., 2020). The tolerance to low temperature can be promoted, for instance, by the synthesis of very-long-chain-fatty-acids (FA with a number of Carbon atoms >18) or by increasing the level of unsaturation. The modification of the membrane fluidity through a desaturation process is a primary mechanism adopted by plants to adapt to low temperatures, making the membrane more fluid, stable and permeable (Lim et al., 2017; Zhukov and Shumskaya, 2020). In apple fruit affected by storage disorder (such as superficial scald), a variation of the lipid accumulation and expression of genes related to lipid metabolism have been proposed (Mellidou et al., 2014; Leisso et al., 2015; Gapper et al., 2017; Busatto et al., 2018). In ‘Honeycrisp’ apple cultivar, for instance, tissues affected by soggy breakdown showed a 35 fold less accumulation of linoleic acid with regards to non-affected tissues. In our survey, we focused our attention on the differential accumulation of fatty acids between the two cultivars and the two postharvest conditions. In the lipid-metabolite assessment we performed, the sample of ‘Granny Smith’ treated with 1-MCP showed a higher accumulation of four specific types of fatty acids, such as palmitic acid, oleic acid, linoleic acid and linolenic acid, which are the most abundant and predominant fatty acids in plants (He and Ding, 2020). Especially the last three presented an increasing level of desaturation, and the degree of unsaturation of the chloroplast phosphatidylglycerol is one of the most important physiological responses to cold treatment, also through a possible activation of the jasmonate pathway. Farmer and Ryan (1992) illustrated that the concentration of linolenic acid, a precursor of jasmonate, can increase during different types of biotic and abiotic stresses. Indeed, one of the first metabolic regulation of the jasmonic acid biosynthesis, is the hydrolyzation of phospholipids, performed by the phospholipase, with the subsequent release of linolenic acid from cell membrane (Turner et al., 2002). Type A and D of phospholipase have been indicated to be actively involved in the jasmonate biosynthesis during stresses in *Arabidopsis* and tomato, respectively. The possible involvement of jasmonates in the control of superficial scald through the exogenous application of 1-MCP was furthermore supported by the identification of a *LOX* gene with a similar transcript accumulation pattern (**Supplementary Figure 6**). Lipoxygenase (LOX) is in fact involved in the jasmonate pathway by coordinating the oxygenation of fatty acids, in particular the 13-LOX type (Turner et al., 2002). The expression of this gene was moreover more relevant in the asymptomatic tissues, especially in those treated with 1-MCP. It has also been reported that methyl-jasmonate can protect fruits and vegetables from chilling injuries, improving plant response to cold (González-Aguilar et al., 2000; Ding et al., 2002). In this scenario, the application of 1-MCP induced the accumulation of unsaturated fatty acids that can contribute to the protection mechanism against cold damage and in turn preventing the development of this disorder. An increased accumulation of unsaturated fatty acids, especially C18:3 linolenic acid, through a transgenic approach, successfully enhanced the tolerance to cold temperature in tobacco and tomato (Kodama et al., 1994; Domínguez et al., 2010). In addition to this mechanism, it is also worth noting that C18 UFAs due to their chemical structure can exert also an active antioxidant role, directly exhausting ROS molecules, such as superoxide anion and hydrogen peroxide (Lim et al., 2017; He and Ding, 2020). The other cultivar ‘Ladina’ was also distinguished by a distinct lipid profile, showing for the three samples a higher accumulation of saturated VLCFAs, such as arachidic acid (C20:0), behenic acid (C22:0) and lignoceric acid (C24:0) (**Figures 3B, D, F**). Although saturated VLCFAs seem to be involved in biotic and abiotic stresses such as cold, drought and oxidative stresses, the entire mechanism of action still remains poorly characterized (De Bigault Du Granrut and Cacas, 2016; Zhukov and Shumskaya, 2020). Nevertheless, in other systems *i.e.* during necroptosis in mammalians, an accumulation of saturated VLCFAs was observed by Parisi et al. (2019), which demonstrated that the membrane integrity can be altered more rapidly by lignoceric acid (C24) than palmitic acid (C16), increasing therefore the membrane permeabilization and interfering with the membrane microdomain. In light of these observations, the two cultivars appear to be distinguished by a distinct response to cold, with ‘Granny Smith’ inducing the biosynthesis of unsaturated fatty acids, while ‘Ladina’ promoting the accumulation of VLCFAs, possibly related to an increased membrane destabilization leading to an induction of the superficial scald symptoms, but more experimental evidence are still needed. The active transcriptional reprogramming deputed to the reorganization of the lipid metabolic array in favor of unsaturated fatty acids, was additionally validated by the lipid based interactome (**Figure 8B**). The interactome analysis showed that palmitic acid and the derived unsaturated fatty acids (oleic, linoleic, and linolenic fatty acids) were more positively correlated with the DEGs than the other class of saturated fatty acid, with a fold change spanning from 2.67 to 27.60, providing valuable support about the active transcription reprogramming stimulated by 1-MCP in the biosynthesis of unsaturated type of fatty acids.

### 4.3 The genome-wide RNA-Seq survey reveals a specific transcription re-programming related to the onset of the chilling injury and the mechanism of action of the two scald-preventing postharvest strategies

The overall transcription profile was hierarchically grouped into four clusters, organized according to the types of response played by the fruit regarding the development of the superficial scald. The first two groups (clusters A and B) were represented by DEGs highly expressed in symptomatic samples (GS_RA and the three samples of ‘Ladina’), with cluster B showing a lower expression in ‘Ladina’ compared to cluster A. The KEGG pathway analysis and the GO enrichment investigation highlighted a change in the activity of a series of common processes for both cultivars. Amongst the most enriched KEGG pathways, we found genes involved in response to lipids, fatty acids metabolic process and cellular response to cold, validating the metabolic scenario assessed in this work. Nevertheless, the secondary metabolites and the biosynthesis of phenylpropanoids were the most enriched GO biological processes. In the GO enrichment analysis, it was also identified a gene set involved in the farnesyl diphosphate mevalonate pathway. The farnesyl diphosphate is a precursor of α-farnesene, originally synthesized along the mevalonate pathway and associated with the development of this disorder. The two clusters also shared a pathway related to the metabolism of glutathione, a peptide with important antioxidant function. The abiotic stress related to the low temperature applied during storage can stimulate an increased production of ROS, which can be in fact mitigated by a scavenging mechanism coordinated by both enzymatic and non-enzymatic processes, through the detoxification of H_2_O_2_ into H_2_O and O_2_ coordinated by the action of catalase (CAT) and the ascorbate-glutathione cycle (Martinez et al., 2016; Lee et al., 2019) to maintain the cellular redox state. With the onset of superficial scald, Rao et al. (1998) observed a high production of H_2_O_2_ paralleled by a consequent increased expression of glutathione reductase, especially in susceptible apple cultivars.

The other two clusters (C and D), where instead composed by DEGs more expressed in the two sample of GS with asymptomatic skin due to the preventing action of 1-MCP or storage at hypoxia atmosphere. Between the two, cluster C grouped the DEGs highly expressed in the GS sample treated with 1-MCP. In accordance with the metabolite profiling, this cluster was characterized by enriched pathways almost exclusively involved in the metabolism of fatty acids, fatty acid elongation and the biosynthesis of unsaturated fatty acids. These pathways, further represented by enriched terms in the GO biological process dictionary, validated as the application of 1-MCP contributed to the control of superficial scald through a molecular and metabolic reprogramming of the fatty acids and lipid constitution. The GO enrichment analysis, moreover, elucidated that this mechanism is accomplished through three major processes: 1) biosynthesis of unsaturated types of fatty acids; 2) assembly of very long chain fatty acids, and 3) faster turnover of glycerophospholipids, cutin and wax biosynthesis. Glycerophospholipids are the major constituent of the cellular internal membrane, providing stability and fluidity, and their biosynthesis and promotion are essential for the plant to resist to long-term persistence of low temperature (Zheng et al., 2016). It is also worth noting the identification of genes involved in the biosynthesis of cutin and wax. The cuticle of fruit, composed by cutin (C16-C20, long chain fatty acids and glycerol) and wax (very long chain fatty acids, non-polar hydrocarbon and derivates), plays a vital role in the protection of fruit to external stimuli from the surrounding environment, especially drought and cold (Hen-Avivi et al., 2014). Particularly during cold storage, important modification in the fruit cuticle composition have been identified, even in ‘Granny Smith’, where long exposure to low temperature during storage resulted in an increased amount of wax (Lara et al., 2019). Cluster D, defined by genes expressed in asymptomatic tissues of ‘Granny Smith’ stored with low oxygen atmosphere, showed, instead, a typical physiological response to hypoxia. The KEGG and GO enrichment analysis identified the photosynthetic processes, such as photosynthesis light harvesting, light reaction and chlorophyll biosynthesis process, as the most enriched pathway. A similar situation was previously reported by Leisso et al. (2016), where storage at low oxygen concentration stimulated the expression of genes involved in chlorophyll catabolism and chloroplast metabolism. This metabolism can be interpreted as a last attempt to increase the energy needed to maintain the overall cell integrity, before a decrease in energy production due to initiation of fermentation processes starts (Mellidou et al., 2014; Cukrov et al., 2016). Parallel to this, the interrogation of the KEGG database also revealed a high enrichment of elements involved in carbon metabolism, glycolysis and pyruvate metabolism. This finding is consistent with the general metabolic reprogramming occurring during hypoxia or anoxia conditions. In a situation of oxygen deprivation, a metabolic reconfiguration related to an oxidative stress is activated due to a disrupted central carbon metabolism. In this situation, the ATP production shifted from a mitochondrial aerobic respiration to an anaerobic fermentation, and since during hypoxia the tricarboxylic acid cycle (TCA) is inhibited, the ATP is provided by glycolysis and the fermentation of pyruvate to ethanol through the pyruvate decarboxylase and alcohol dehydrogenase process (Mellidou et al., 2014). The transition in the mode of respiration with the shift from aerobic to anaerobic (fermentation) was here confirmed by the assessment of the volatilome that highlighted a higher accumulation of ethanol and its precursor acetaldehyde in the samples stored in hypoxia condition (LO) for both cultivars. The two volatiles were also distinguished by a higher proportion of positive correlations with the DEGs in the volatilome based interactome, with a fold change of 3.75 to 68 with regards to the negative correlation (**Figure 8C**). This highlight that these metabolites are actively synthesized following specific storage conditions, such as low oxygen. It is also worth noting the metabolic pattern of these two metabolites, which resulted much more accumulated in ‘Ladina’ than in ‘Granny Smith’, already in the control sample (RA). The preventive mechanism of low oxygen on the development of superficial scald has been associated to different modes of action played by ethanol, from inhibitor of ethylene to antioxidant molecule (Lurie and Watkins, 2012), although not completely elucidated yet. However, this role cannot be completely generalized, since samples of ‘Ladina’, despite the high accumulation of ethanol, showed severe superficial scald symptoms, strengthening the role of the different genetic background in the control of this disorder. Moreover, the excessive accumulation of ethanol can lead to a fruit quality decay for the possible generation of off-flavor compounds (Cukrov et al., 2016). The production of ATP through a fermentation process represents, moreover, a metabolic step towards the acclimation process to sustain the survival in unfavorable situation (Loreti et al., 2018). The machinery leading to acclimation was here supported by the finding of three DEGs specifically up-regulated in the GS_LO sample and assigned to the VII subgroups of *ERF* genes ortholog of the *Arabidopsis RAP2.12.* The synthesized protein, inactively stored in the plasma membrane in normoxic condition, is translocated to the nucleus after the establishment of hypoxia, triggering a physiological reprogramming in response to oxygen deprivation. In normoxia situation this element is degraded through the N-end rule pathway leading to protein degradation (Gibbs et al., 2011; Licausi et al., 2011). During hypoxia, the *ERF-VII* genes coordinate the acclimation process necessary to contrast the effect of low oxygen by binding HRPE (hypoxia response promoter elements) present in the promoter regions of genes playing crucial roles during anaerobiosis, such as *pyruvate decarboxylase (PDC)* and *alcohol dehydrogenase (ADH)* (Hess et al., 2011). This also finds consistency with the data presented here, where the expression of three *ERF-VII* genes were positively correlated with the expression levels of three *alcohol dehydrogenases* (LOC103432769, LOC103435799 and LOC103400521) and a *pyruvate decarboxylase* (LOC103425929), with Pearson’s correlation values spanning from 0.77 to 0.99. In this scenario, ERF-VII proteins play a master regulatory role in reconfiguring the central carbon metabolism towards the activation of fermentative processes in oxygen depletion situation also in apple stored at low oxygen atmosphere for the control of superficial scald. It is also worth noting that among the VOCs assessed during this survey, the ethylene profile was also inspected. This compound is a primary hormone controlling the fruit ripening, but it has also recognized as a major driver involved in the control of the superficial scald. However, in this survey the interactome based on ethylene did not find any correlation with DEGs. This might lead to hypothesize that ethylene could be not directly involved in the physiological control of this disorder, therefore the ethylene competing strategy applied during postharvest, such as 1-MCP or LO, would interfere with superficial scald by modulating other pathways involved in this regulation, such as phenolic or lipid metabolism.

Beside the identification of reprogramming events related to specific metabolites, the global RNA-Seq survey revealed a distinct transcriptomic signature between the two cultivars. Among the set of DEGs, the two most enriched groups of genes showing a higher expression in ‘Ladina’ then in ‘Granny Smith’ were represented by *Mal d 1* and *Mal d 1-like* and *DMR6 oxygenase 2-like* genes, both activated in response to different kind of stresses. Although Mal d 1 and Mal d 1-like family protein are known to be one of the most important class of allergens in apple (Ahammer et al., 2017), they belong to the pathogenesis-related proteins of the PR-10 sub-class (Fernandes et al., 2013), which are small proteins (17kDa) with a ribonuclease activity. It has been already reported that these proteins can be induced by different types of stresses, such as: biotic and abiotic factors (*i.e.* cold, drought, oxidative stress), phytohormones (*i.e.* ethylene, jasmonic acid, salicylic acid) and physical damage like wounding (Liu and Ekramoddoullah, 2006; Agarwal and Agarwal, 2013; Fernandes et al., 2013). These proteins are also highly accumulated in the peel of apples (Marzban et al., 2005; Pagliarani et al., 2013), and their gene expression levels increased during postharvest storage, in response to stress (cold, wounding and pathogen infection *i.e. Venturia inaequalis*) and in a cultivar dependent manner (Poupard et al., 2003; Matthes and Schmitz-Eiberger, 2009; Schmitz-Eiberger and Matthes, 2011; Gusberti et al., 2013; Savazzini et al., 2015). Despite the fact that the exact mechanisms of action of this protein remain poorly characterized, based upon these observations it has been proposed that Mal d 1, as PR-10 proteins in general, may be involved in the response mechanism against biotic and/or abiotic stresses (Poupard et al., 2003; Agarwal and Agarwal, 2013; Fernandes et al., 2013; Gusberti et al., 2013; Zhang et al., 2021). Similarly, but with an opposite role, *DMR6-oxygenase* elements were also more expressed in ‘Ladina’. *DMR6* is a susceptibility gene that negatively affects plant defense (de Toledo Thomazella et al., 2021) and utilizing molecular O_2_ can coordinate the oxidization process of different substrates (Van Damme et al., 2008; Zeilmaker et al., 2015). Despite the fact that *DMR6* gene is mainly expressed during pathogen infection, it has been proposed to be also involved during abiotic stresses (de Toledo Thomazella et al., 2021). The different magnitude in gene expression between these two cultivars (with an average fold change of about 6), suggests a specific transcriptomic signature related to a distinct genetic background in this process, and we hypothesize that the higher expression of genes such as *Mal d 1-like* or *DMR6-like* might have a role in the promotion of the susceptibility of ‘Ladina’ to superficial scald, even when the strategies known to interfere with this phenomenon were applied.

These findings elucidate the regulatory mechanism of the postharvest disorder superficial scald in two apple cultivars distinguished by a different behavior and underlined the relevance of these types of investigation to better characterize the postharvest physiology of each specific apple cultivar. Although superficial scald is regulated by a common physiological process among different cultivars, its regulation and mode of action of the currently known preventing strategies seems to be more cultivar specific. This is also supported by the observation that beside the role of 1-MCP and LO to efficiently control the production of ethylene in both apple cultivars, the efficacy in controlling the development of superficial scald was very different and genetically dependent. Given the continued demand and interest for novel cultivars suitable for more sustainable horticulture or simply characterized by superior fruit quality, a more informed study of their postharvest performance is essential to reduce costs, energy, and food waste to enable better food security for apples.

## Data availability statement

The RNA-Seq raw data are freely available at the GEO database (accession number: GSE216082).

## Author contributions

FC, AZ and NB conceived the work and designed the study. LV, FP performed the molecular experiments. SS, AZ, AB and SB-S collected fruit and performed the postharvest storage, FP, IK, FB, UV and DM performed the metabolite analysis, FP and LV performed the data analysis, FC, FP, LV and NB drafted the manuscript. All authors contributed to the article and approved the submitted version.
