# Supplementary figures and images for: Comparative transcriptome and metabolite survey reveal key pathways involved in the control of the chilling injury disorder superficial scald in two apple cultivars, ‘Granny Smith’ and ‘Ladina’
Source: Front Plant Sci. 2023 Apr 20;14:1150046. doi: 10.3389/fpls.2023.1150046 (PMC10157158; doi:10.3389/fpls.2023.1150046)

## Slide 1
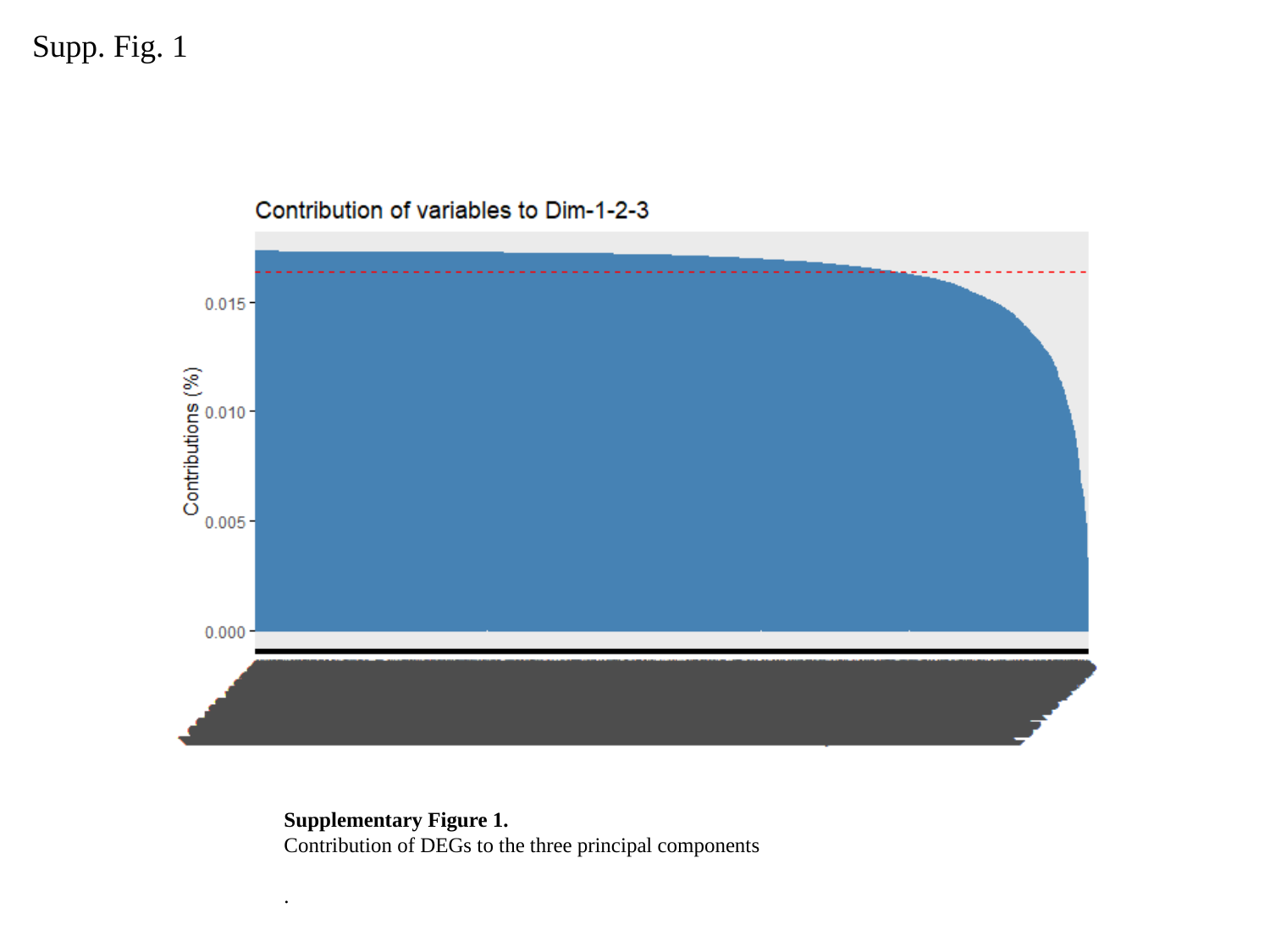

Supp. Fig. 1
Supplementary Figure 1.
Contribution of DEGs to the three principal components
.

Supplement: Supplementary file 1 [file Presentation_1.pptx]
