# Supplementary material for: Comparative transcriptome and metabolite survey reveal key pathways involved in the control of the chilling injury disorder superficial scald in two apple cultivars, ‘Granny Smith’ and ‘Ladina’
Source: Front Plant Sci. 2023 Apr 20;14:1150046. doi: 10.3389/fpls.2023.1150046 (PMC10157158; doi:10.3389/fpls.2023.1150046)
Supplement: Supplementary file 2 [file Presentation_2.pptx]

## Slide 1
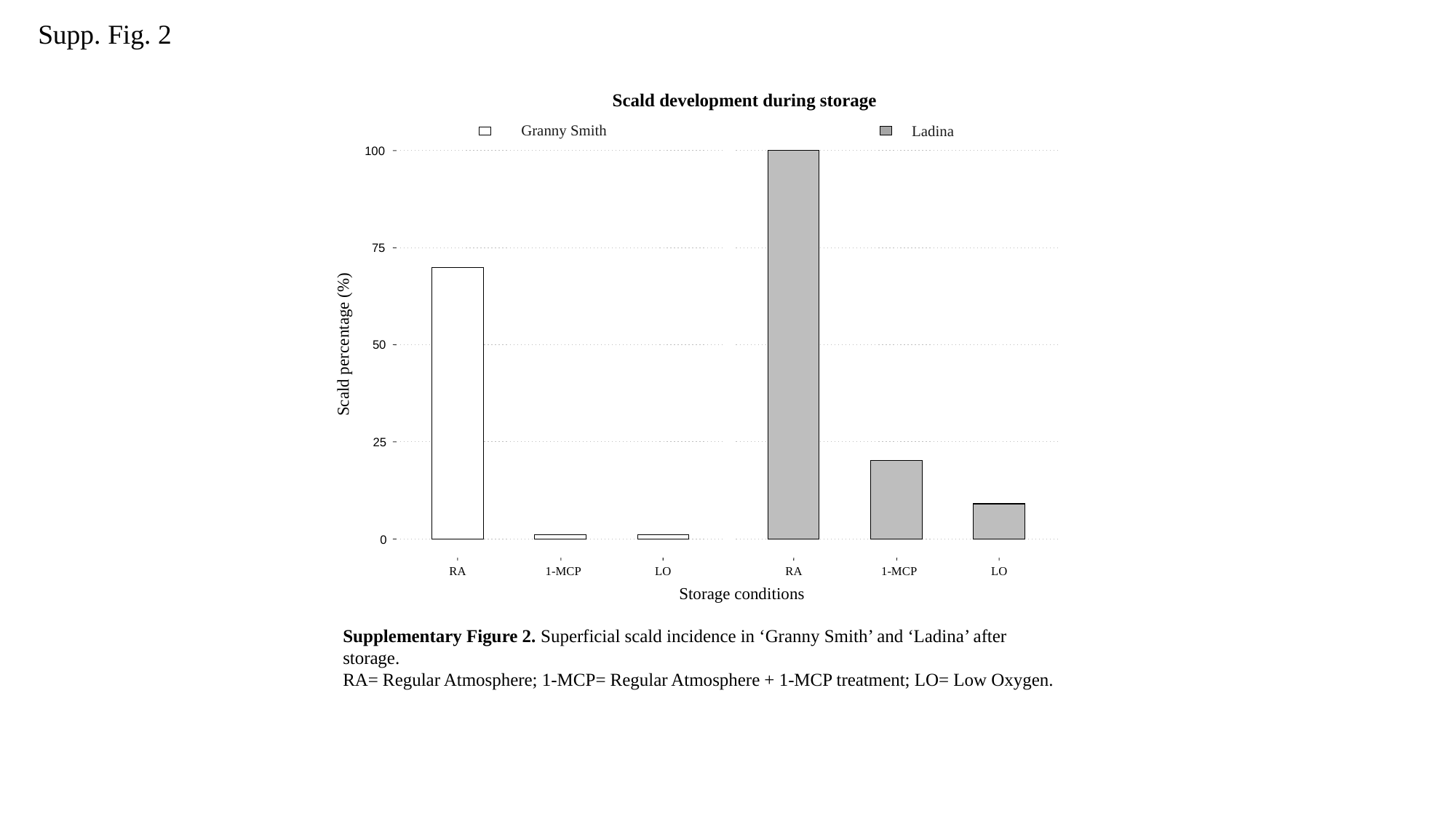

Supp. Fig. 2
Scald development during storage
Granny Smith
Ladina
100
75
Scald percentage (%)
50
25
0
RA
1-MCP
LO
RA
1-MCP
LO
Storage conditions
Supplementary Figure 2. Superficial scald incidence in ‘Granny Smith’ and ‘Ladina’ after storage.
RA= Regular Atmosphere; 1-MCP= Regular Atmosphere + 1-MCP treatment; LO= Low Oxygen.
