# Supplementary material for: Comparative transcriptome and metabolite survey reveal key pathways involved in the control of the chilling injury disorder superficial scald in two apple cultivars, ‘Granny Smith’ and ‘Ladina’
Source: Front Plant Sci. 2023 Apr 20;14:1150046. doi: 10.3389/fpls.2023.1150046 (PMC10157158; doi:10.3389/fpls.2023.1150046)
Supplement: Supplementary file 3 [file Presentation_3.pptx]

## Slide 1
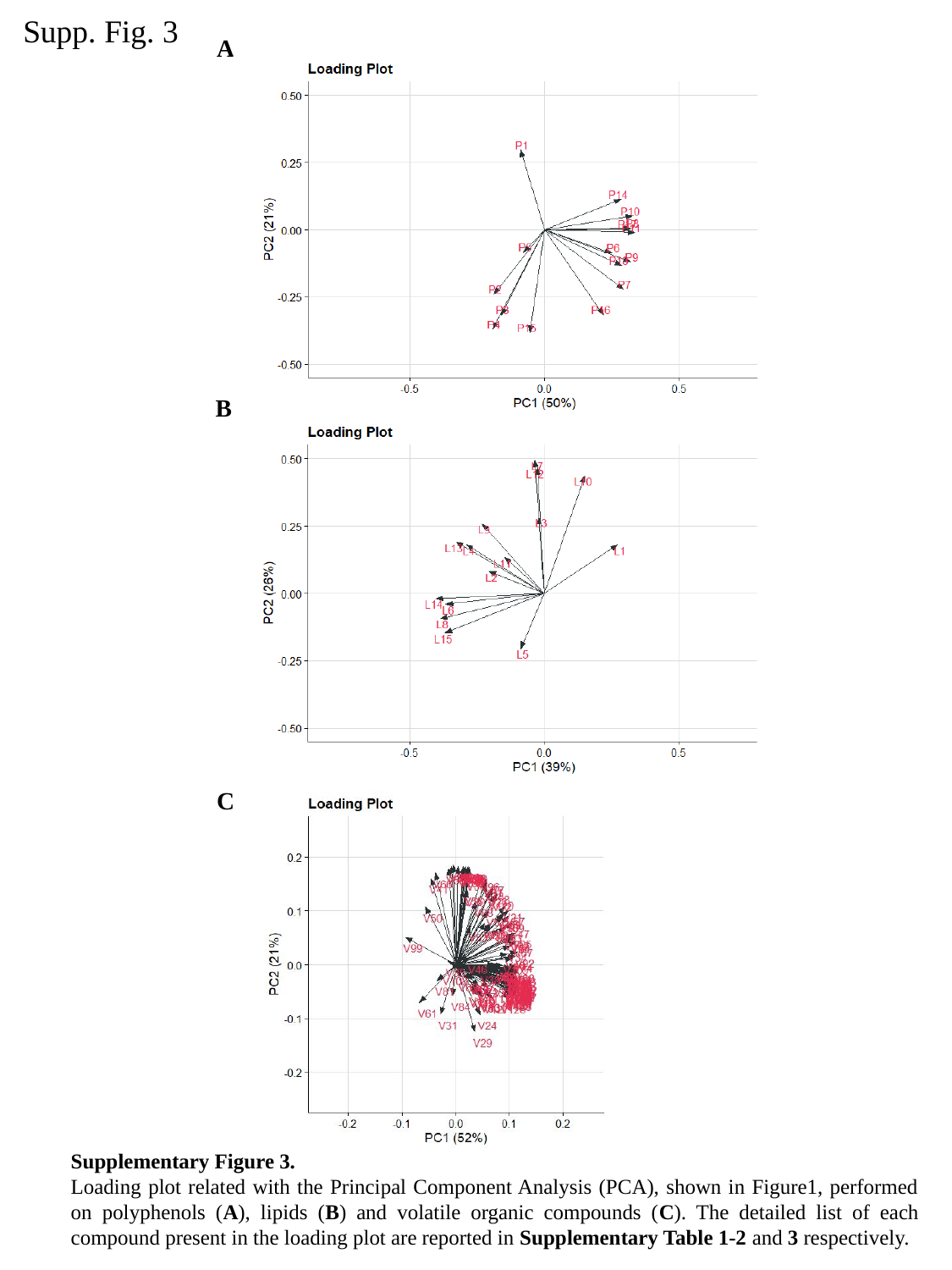

Supp. Fig. 3
A
B
C
Supplementary Figure 3.
Loading plot related with the Principal Component Analysis (PCA), shown in Figure1, performed on polyphenols (A), lipids (B) and volatile organic compounds (C). The detailed list of each compound present in the loading plot are reported in Supplementary Table 1-2 and 3 respectively.
