# Supplementary material for: Comparative transcriptome and metabolite survey reveal key pathways involved in the control of the chilling injury disorder superficial scald in two apple cultivars, ‘Granny Smith’ and ‘Ladina’
Source: Front Plant Sci. 2023 Apr 20;14:1150046. doi: 10.3389/fpls.2023.1150046 (PMC10157158; doi:10.3389/fpls.2023.1150046)
Supplement: Supplementary file 4 [file Presentation_4.pptx]

## Slide 1
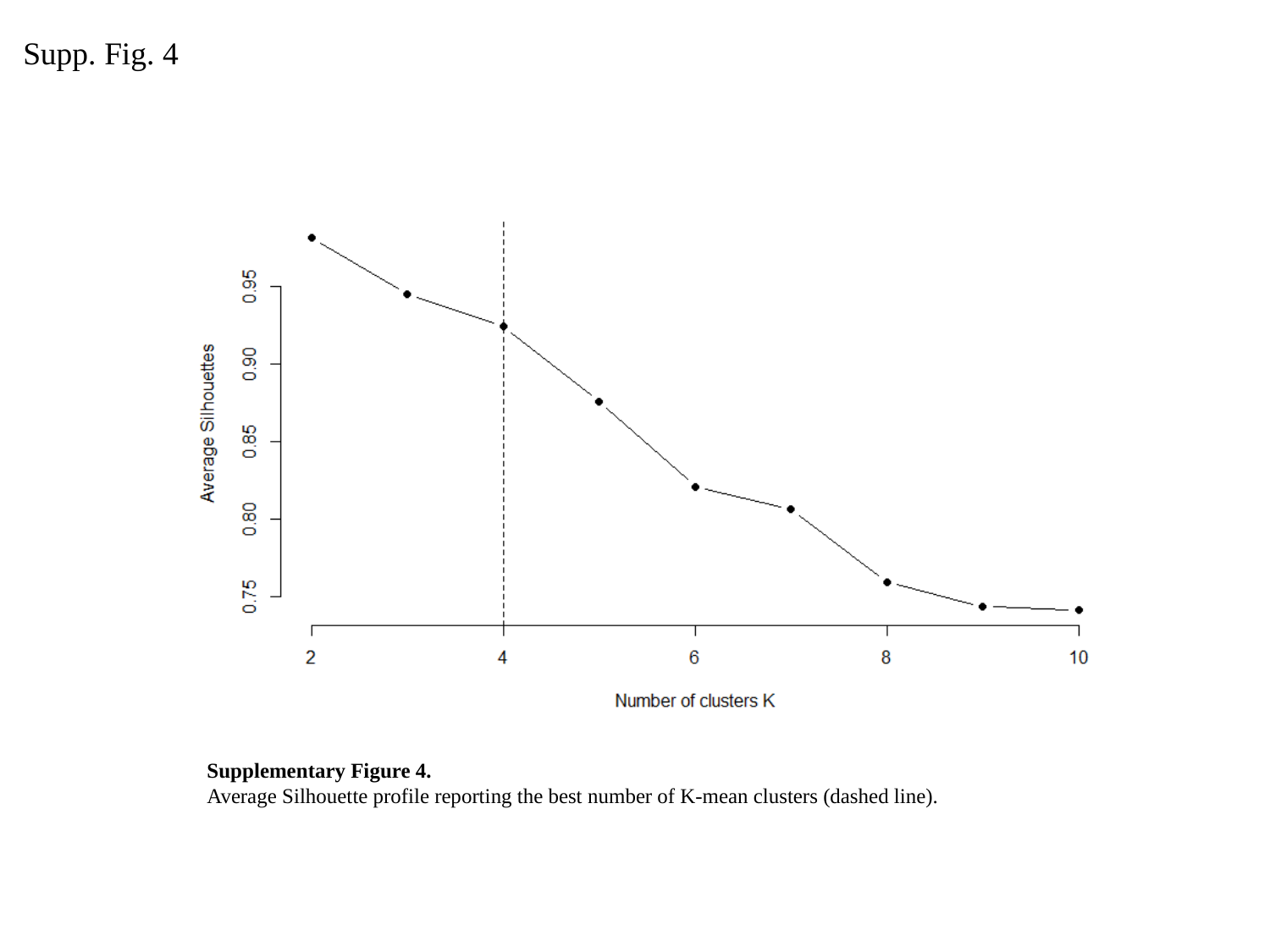

Supp. Fig. 4
Supplementary Figure 4.
Average Silhouette profile reporting the best number of K-mean clusters (dashed line).
