# Supplementary material for: Comparative transcriptome and metabolite survey reveal key pathways involved in the control of the chilling injury disorder superficial scald in two apple cultivars, ‘Granny Smith’ and ‘Ladina’
Source: Front Plant Sci. 2023 Apr 20;14:1150046. doi: 10.3389/fpls.2023.1150046 (PMC10157158; doi:10.3389/fpls.2023.1150046)
Supplement: Supplementary file 5 [file Presentation_5.pptx]

## Slide 1
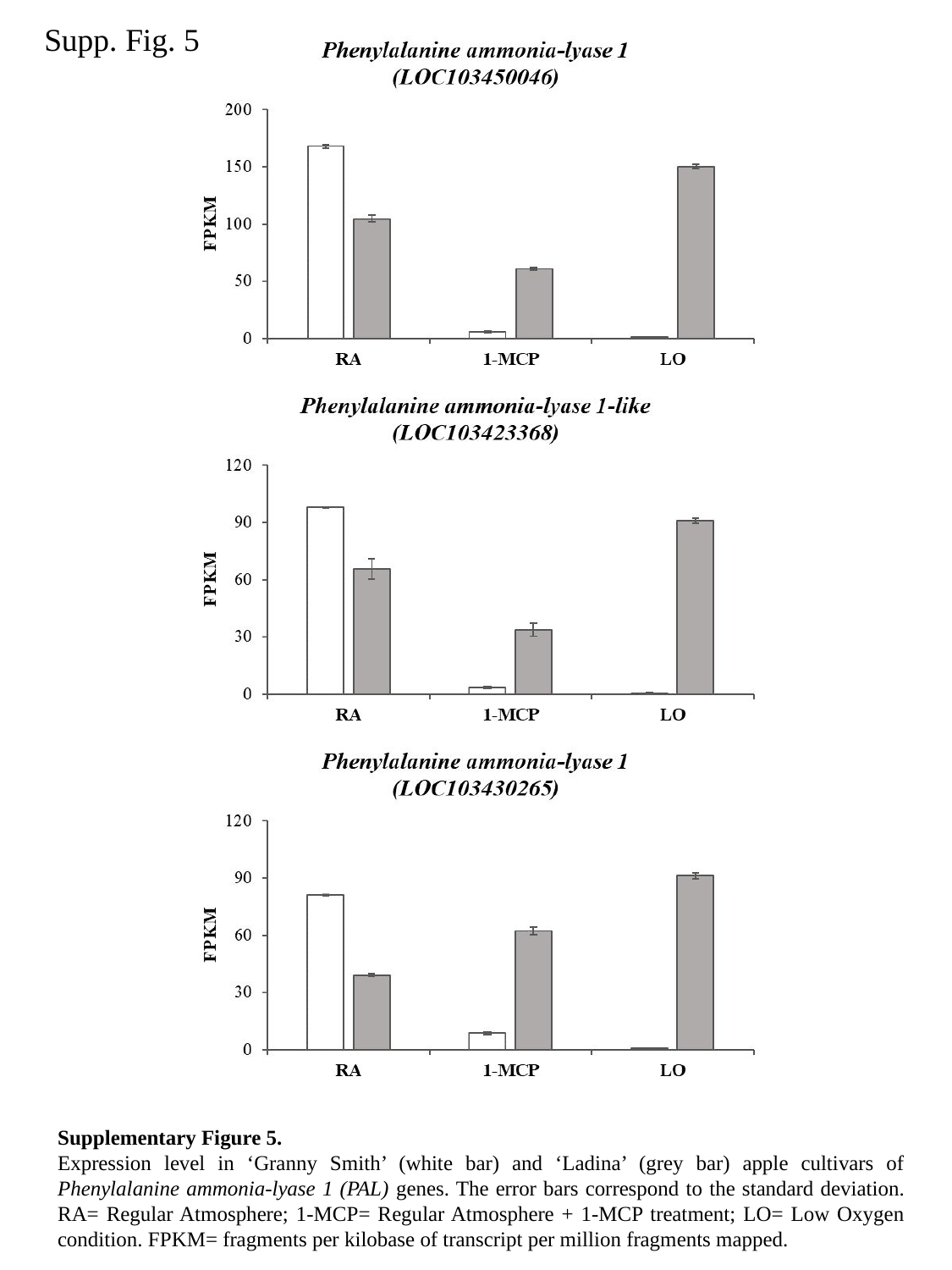

Supp. Fig. 5
Supplementary Figure 5.
Expression level in ‘Granny Smith’ (white bar) and ‘Ladina’ (grey bar) apple cultivars of Phenylalanine ammonia-lyase 1 (PAL) genes. The error bars correspond to the standard deviation. RA= Regular Atmosphere; 1-MCP= Regular Atmosphere + 1-MCP treatment; LO= Low Oxygen condition. FPKM= fragments per kilobase of transcript per million fragments mapped.
