# Supplementary material for: Comparative transcriptome and metabolite survey reveal key pathways involved in the control of the chilling injury disorder superficial scald in two apple cultivars, ‘Granny Smith’ and ‘Ladina’
Source: Front Plant Sci. 2023 Apr 20;14:1150046. doi: 10.3389/fpls.2023.1150046 (PMC10157158; doi:10.3389/fpls.2023.1150046)
Supplement: Supplementary file 6 [file Presentation_6.pptx]

## Slide 1
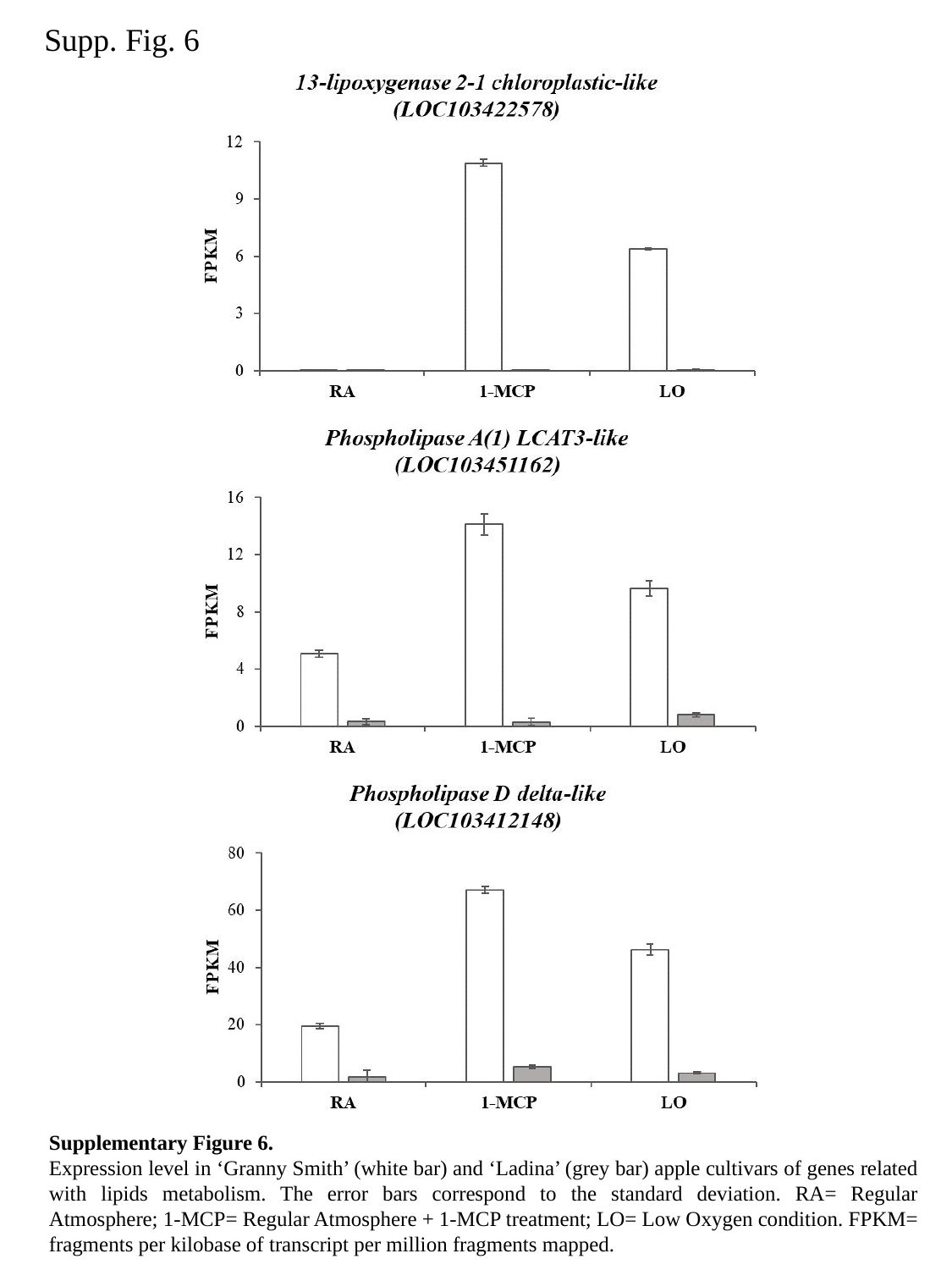

Supp. Fig. 6
Supplementary Figure 6.
Expression level in ‘Granny Smith’ (white bar) and ‘Ladina’ (grey bar) apple cultivars of genes related with lipids metabolism. The error bars correspond to the standard deviation. RA= Regular Atmosphere; 1-MCP= Regular Atmosphere + 1-MCP treatment; LO= Low Oxygen condition. FPKM= fragments per kilobase of transcript per million fragments mapped.
